# Stabile fluoro-benzene-based spacer for lead-free Dion–Jacobson perovskites†

**DOI:** 10.1039/d2ra07675f

**Published:** 2023-01-04

**Authors:** Chih Shan Tan

**Affiliations:** a Institute of Electronics, National Yang Ming Chiao Tung University Hsinchu 30010 Taiwan cstan@nycu.edu.tw

## Abstract

Two-dimensional perovskite materials have been investigated as potential candidates for next-generation-wide band gap devices and lead-based perovskites are the most common materials within two-and three-dimensional structures due to their superior optoelectronic properties. Nevertheless, the stability and toxic element issues are the two significant shortcomings of device commercialization. The fluoro-benzene-based divalent ammonium spacer cations and replacing Zn^2+^ with Pb^2+^ will improve the two-dimensional perovskite stability. These stable lead-free wide band gap two-dimensional structures have better carrier mobility at high-temperature regions. Therefore, lead-free two-dimensional perovskites might be suitable for higher temperatures optoelectronic applications.

## Abstract 2

Due to their superior optoelectronic properties, two-dimensional and lead-free perovskite materials have been investigated as potential candidates for wide band gap devices.

## Graphical abstract

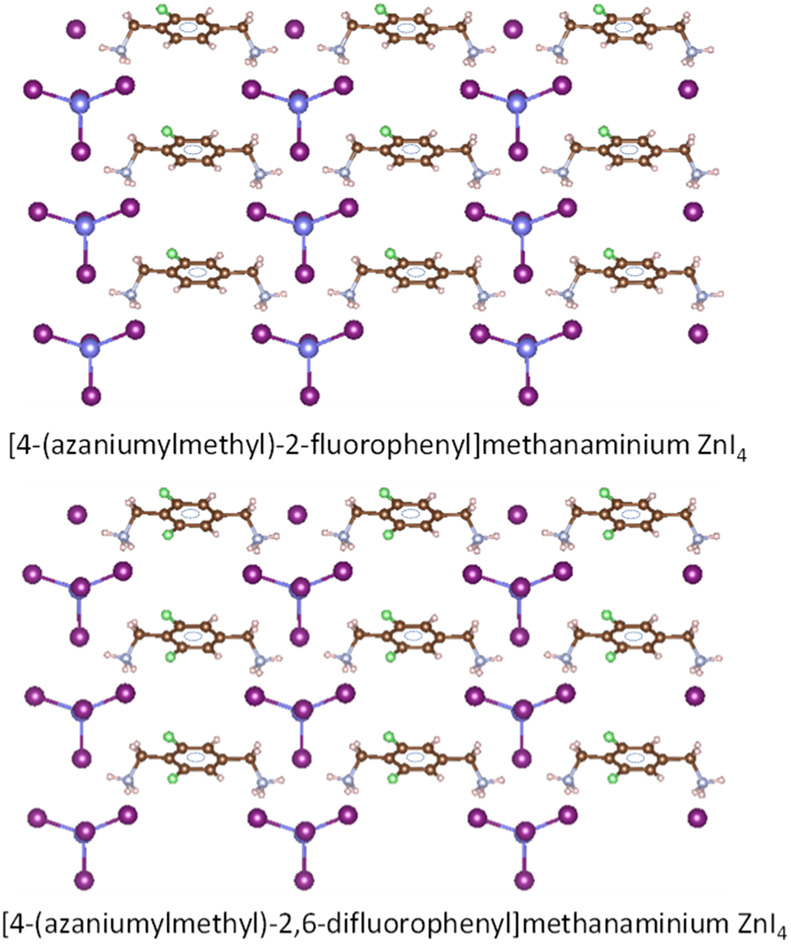

## Introduction

Two-dimensional inorganic materials, such as hBN, MoS_2_, BP, graphene, and TaS_2_, are widely discovered for their unusual optoelectronic properties due to their unique layered structures within van der Waals gaps in device applications.^1–3^ The two-dimensional inorganic materials usually grow by the metal–organic chemical vapor deposition (MOCVD) method, and it is hard to get a uniform thin film within a large area.^4,5^ The comprehensive research on two-dimensional materials has extended toward organic–inorganic hybrid semiconductors, and it might be easy to fabricate a uniform thin film in a large area by spin coating or blading. Recently, two-dimensional organic–inorganic halide perovskites (OIHPs) have been investigated in the types of Ruddlesden–Popper (RP) and Dion–Jacobson (DJ) structures.^6–9^ For the RP OIHPs, the organic ammonium cation spacer separates the perovskite structure and forms van der Waals gaps between the spacers. For the DJ OIHPs, the organic cation spacer separates the layered perovskite and connects the two layers by two diammonium cations. As a result, there is a van der Waals gap difference between RP and DJ OIHPs,^9^ and the DJ OIHPs, without the van der Waals gaps, have better structural stability by avoiding the layer slide issue.

The van der Waals gap within the RP OIHPs makes it easy for the layers to slide, and might cause the phase to be unstable. Also, the structure is hard for the electrons and holes to move and worsen the optoelectronic properties.^7^ Therefore, the DJ OIHPs without the van der Waals gap might be suitable for optoelectronic device applications, and there has been much research discussion on the DJ OIHPs.^10–16^ The DJ OIHPs have opened a new extension research field with better stability and optoelectronic properties for the two-dimensional lead perovskites and are more complicated than the typical perovskite with the ABX_3_ structure. The divalent ammonium spacer cations have different aromatic rings and functional groups between the two ammonium cations on the head and tail. The cation spacer, with the π electron, will improve the DJ OIHP structure stability, and the variation of the π electron condition might influence the two-dimensional crystal stability. Thus, the structure of divalent ammonium spacer cations needs further investigation. In this study, the different forms of the benzene rings and functional groups for the divalent ammonium spacer cations of the DJ OIHPs are screened using density functional theory (DFT) calculations.

The origin setup is to find directions for improving the structural stability and optoelectronic properties by changing the length and types of functional groups of the divalent ammonium spacer cations. By the phonon density of state calculation, the [4-(azaniumylmethyl)phenyl]methanaminium PbI_4_ has better dynamic stability than benzene-1,4-bis(aminium) PbI_4_ and 2-[4-(2-azaniumylethyl)phenyl]ethan-1-aminium PbI_4_. Here, a suitable length of the divalent ammonium spacer cations was found. With different halides and methyl added to the benzene of the [4-(azaniumylmethyl)phenyl]methanaminium PbI_4_, the [4-(azaniumylmethyl)-2-fluorophenyl]methanaminium PbI_4_ and the [4-(azaniumylmethyl)-2,6-difluorophenyl]methanaminium PbI_4_ have better dynamic stability. This indicates that the fluoro group could stabilize the DJ OIHPs in the divalent ammonium spacer cations. Finally, the Mg^2+^, Ca^2+^, Sr^2+^, Zn^2+^, Eu^2+^, and Yb^2+^ are used to replace the Pb^2+^ cation of the [4-(azaniumylmethyl)-2-fluorophenyl]methanaminium PbI_4_ and the [4-(azaniumylmethyl)-2,6-difluorophenyl]methanaminium PbI_4_. The phonon calculation results showed that Zn^2+^ could be a candidate to form lead-free DJ OIHPs with better structural dynamic stability.

## Results and discussion

**Fig. 1:**
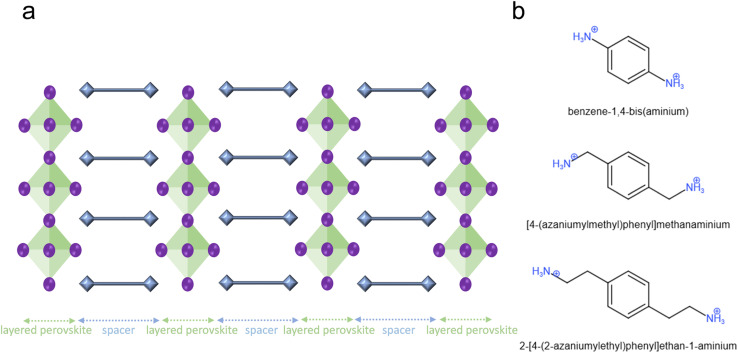
The two-dimensional Dion–Jacobson perovskite and different spacer cations. (a) The 2D DJ layer structures are composed of a diammonium organic spacer layer (blue) and a 2D metal halide perovskite layer. (b) The typical two-diammonium aromatic spacer cations.

Fig. 1a illustrates the DJ OIHP structure. The green and blue areas are the perovskites and the spacer parts. The blue squares at the two ends of the spacer are the ammonium cations, and the ammonium cations inserted at the A site of ABX_3_ perovskite structure and transform the formula toward spacer cation-BX_4_ perovskite (B = Pb and X = halides). Benzene has three pairs of π electrons, and the ability to move and oscillate π electrons will cause benzene to apply in conductive polymers^9^ and semiconductors. Thus, the cation spacers with the benzene structure should be investigated for splitting the 3D perovskite structure toward the stable 2D perovskite. For the DJ OIHP solar cell, the poly(2,5-dimethylaniline) (PDMA) could be used as the divalent ammonium spacer cations with a 15.6% power conversion efficiency (PCE).^7^

Due to the high PCE of PDMA-based 2D solar cells, the PDMA-related divalent ammonium spacer cations within the DJ OIHP should be discussed. The PDMA is the benzene connected to two methyls symmetrically, and for both methyls, one of the hydrogens is changed to the NH_3_^+^. Indeed, the IUPAC (International Union of Pure and Applied Chemistry) name of PDMA is [4-(azaniumylmethyl)phenyl]methanaminium, and in this paper, the IUPAC name is preferred. The right spacer length of stable DJ OIHP needs to be found. In Fig. 1b, the benzene-1,4-bis(aminium), [4-(azaniumylmethyl)phenyl]methanaminium, and 2-[4-(2-azaniumylethyl)phenyl]ethan-1-aminium were created for discussion. The three divalent ammonium spacer cations were used to build the PbI_4_-based DJ OIHPs for phonon density of the state (DOS) calculations. The imaginary part of the phonon DOS results of the three divalent ammonium spacer cations is shown in Table 1. For the imaginary part of phonon DOS, the lower portion usually means that the structure has better dynamic stability.^17–20^ The imaginary (negative) phonon DOSs offer the phonon transition possibility from normal positive energy states to negative energy states, which will automatically lose bond energy. The more imaginary part of the phonon DOS accompanies more opportunities to lose bond energy by phonon and finally break the crystal structure. In Table 1, the [4-(azaniumylmethyl)phenyl]methanaminium PbI_4_ has the lowest imaginary part of phonon DOS of 2.76%, than benzene-1,4-bis(aminium) PbI_4_ of 6.55%, and 2-[4-(2-azaniumylethyl)phenyl]ethan-1-aminium PbI_4_ of 2.81%, which means that the [4-(azaniumylmethyl)phenyl]methanaminium PbI_4_ has better dynamic stability. According to the phonon calculation, the result helps to realize the reason that the PDMA-based DJ OIHP could be used for a solar cell with better device stability.^7^

**Table 1:** The imaginary part of the phonon DOS of benzene-1,4-bis(aminium) PbI_4_, [4-(azaniumylmethyl)phenyl]methanaminium PbI_4_, and 2-[4-(2-azaniumylethyl)phenyl]ethan-1-aminium PbI_4_

Crystal structure	Imaginary part of phonon DOS (%)
Benzene-1,4-bis(aminium) PbI_4_	6.55
[4-(Azaniumylmethyl)phenyl]methanaminium PbI_4_	2.76
2-[4-(2-Azaniumylethyl)phenyl]ethan-1-aminium PbI_4_	2.81

In Fig. S1 to S5,† the fluoro- (Fig. S1†), chloro- (Fig. S2†), bromo- (Fig. S3†), iodo- (Fig. S4†), and methyl (Fig. S5†) functional groups are added to the benzene circle by replacing the hydron or hydrons of the [4-(azaniumylmethyl)phenyl]methanaminium. For each functional group on the benzene of the [4-(azaniumylmethyl)phenyl]methanaminium structure, the variations are 2, 2-3, 2-5, 2-6, 2-3-6, and 2-3-5-6 sites. After a slight change in the functional groups of [4-(azaniumylmethyl)phenyl]methanaminium, the new divalent ammonium spacer cations are used to form the DJ OIHPs with layered PbI_4_ for the phonon calculations. The related imaginary phonon DOSs are listed in Tables S1 to S5† for the dynamic stability discussion. Fig. S1 and Table S1† show that the fluoro-benzene-based spacer DJ OIHP variations have imaginary phonon DOS values from 0.69% to 2.89%. According to Fig. S2 and Table S2,† the chloro-benzene-based spacer DJ OIHP variations have imaginary phonon DOS values from 1.31% to 4.03%. In Fig. S3 and Table S3,† the bromo-benzene-based spacer DJ OIHP variations have imaginary phonon DOS values from 1.22% to 3.18%. In Fig. S4 and Table S4,† the iodo-benzene-based spacer DJ OIHP variations have imaginary phonon DOS values from 1.30% to 2.88%. In Fig. S5 and Table S5,† the methyl-benzene-based spacer DJ OIHP variations have imaginary phonon DOS values from 1.81% to 3.64%. The details of the methyl-benzene-based spacer DJ OIHPs shown in Fig. S5 and Table S5† have higher imaginary phonon DOS values, this point out that the methyl-benzene-based spacer DJ OIHPs might not be stable. The data also point out that the fluoro-benzene-based spacer DJ OIHPs have the lowest imaginary phonon DOSs of 0.69% and 0.71% for the [4-(azaniumylmethyl)-2,6-difluorophenyl]methanaminium PbI_4_ and the [4-(azaniumylmethyl)-2-fluorophenyl]methanaminium PbI_4_. These two DJ OIHPs are the only two structures, with Pb^2+^, with imaginary phonon DOS values lower than 1% in this research.

The [4-(azaniumylmethyl)-2,6-difluorophenyl]methanaminium PbI_4_ and the [4-(azaniumylmethyl)-2-fluorophenyl]methanaminium PbI_4_ are *para*-divalent ammoniums. The *meta*-divalent ammoniums of the DJ OIHPs are built up for geometry stability discussion. Due to the divalent ammonium cations needed to link the layered PbI_4_, in this research *ortho*-divalent ammonium was not used as the spacer. In Fig. S6,† the [3-(azaniumylmethyl)phenyl]methanaminium PbI_4_ was used for the fluoro-benzene-based DJ OIHP discussion with the *para*-divalent ammoniums. There are ten positions for the fluoro to substitute on the benzene, and the calculated imaginary phonon DOSs are listed in Table S6.† In Table S6,† the [3-(azaniumylmethyl)phenyl]methanaminium PbI_4_, *meta*-divalent ammoniums, have an imaginary phonon DOS of 1.44%, which is lower than the [4-(azaniumylmethyl)phenyl]methanaminium PbI_4_, *para* divalent ammoniums, with imaginary phonon DOS of 2.76%, as shown in Table 1. Therefore, the *meta*-divalent ammoniums have better dynamic stabilities for DJ OIHPs than the *para*, and the fluoro-based *meta*-divalent ammonium spacer should be discussed for further stability improvement. In Table S6,† the imaginary phonon DOS values are 1.44% to 3.35%, which means that the fluoro-based *para*-divalent ammonium spacer DJ OIHPs have the lower imaginary phonon DOS in Table S1.†

**Fig. 2:**
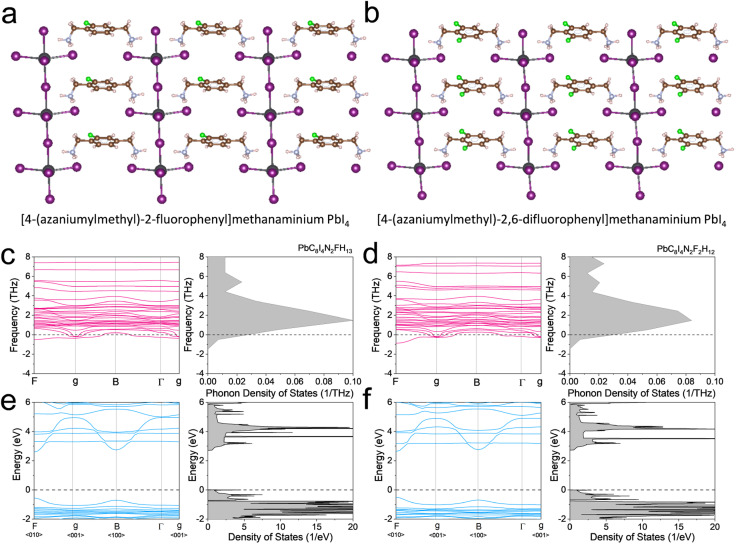
The crystal structure, phonon band diagrams, and electron band diagrams of [4-(azaniumylmethyl)-2-fluorophenyl] methanaminium PbI_4_ and [4-(azaniumylmethyl)-2,6-difluorophenyl] methanaminium PbI_4_. (a and b) The crystal structures (c and d) The phonon band diagrams. (e and f) The electron band diagrams.

**Fig. 3:**
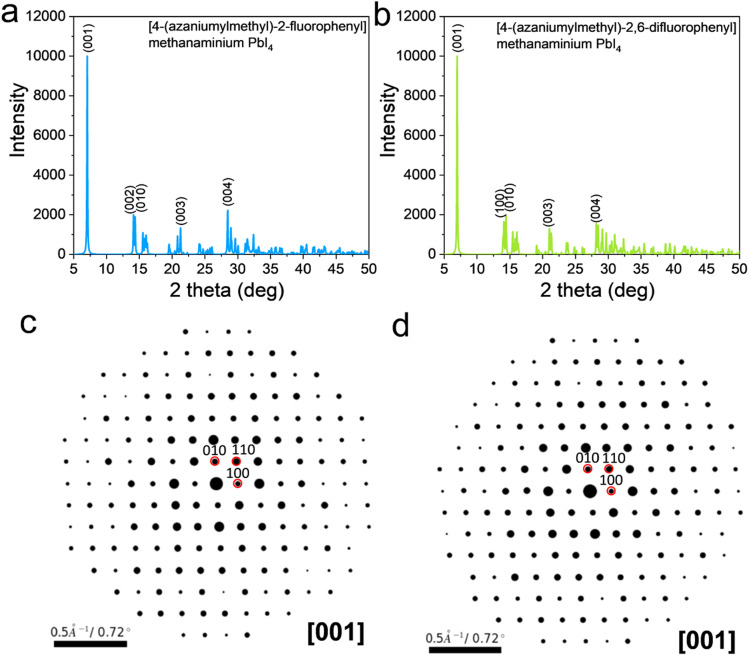
The X-ray and electron diffraction patterns of [4-(azaniumylmethyl)-2-fluorophenyl] methanaminium PbI_4_ and [4-(azaniumylmethyl)-2,6-difluorophenyl] methanaminium PbI_4_. (a and b) The X-ray diffraction patterns. (*λ* = 1.54 Å) (c and d) The electron diffraction patterns (*λ* = 0.025 Å).

The structures of the [4-(azaniumylmethyl)-2-fluorophenyl]methanaminium PbI_4_ and the [4-(azaniumylmethyl)-2,6-difluorophenyl]methanaminium PbI_4_ are shown in Fig. 2a and b. For the [4-(azaniumylmethyl)-2-fluorophenyl]methanaminium PbI_4_, the calculated phonon dispersion and phonon DOS diagrams are shown in Fig. 2c, and there are a few states below the zero dash line. The calculated phonon dispersion and phonon DOS diagrams are shown in Fig. 2d for the [4-(azaniumylmethyl)-2,6-difluorophenyl]methanaminium PbI_4_, and few states are under zero. The band structure and electron DOS diagrams of the [4-(azaniumylmethyl)-2-fluorophenyl]methanaminium PbI_4_ and [4-(azaniumylmethyl)-2,6-difluorophenyl]methanaminium PbI_4_ are shown in Fig. 2e and f. Both of them have the same indirect band gaps of 2.72 eV. The two structures have one fluoro difference in benzene within the spacer, as shown in Fig. 2a and b. The phonon band diagrams differ slightly, as shown in Fig. 2c and d, but the electron band diagrams have no variation, as shown in Fig. 2e and f. The calculated XRD of the [4-(azaniumylmethyl)-2-fluorophenyl]methanaminium PbI_4_ and [4-(azaniumylmethyl)-2,6-difluorophenyl]methanaminium PbI_4_ are shown in Fig. 3a and b. These XRD patterns are nearly identical and could offer a reference for future experimental analysis. As the wavelength of the electron (0.025 Å) is shorter than that of the X-ray (1.54 Å), the electron diffraction patterns, with [001] zone axis, of the two DJ OIHPs were calculated and shown in Fig. 3c and d. The one fluoro difference is also hard to find in the electron diffraction patterns.

Although the flouro added on benzene could stabilize the DJ OIHP, the lead cation inside the DJ OIHP still had an environmental issue. The lead-free DJ OIHP should be an ideal two-dimensional material for future commercialized devices. Here, the Mg^2+^, Ca^2+^, Sr^2+^, Zn^2+^, Eu^2+^, and Yb^2+^ cations are used to replace the Pb^2+^ cations in the [4-(azaniumylmethyl)-2-fluorophenyl]methanaminium PbI_4_ and [4-(azaniumylmethyl)-2,6-difluorophenyl]methanaminium PbI_4_, for the dynamic stability and optoelectronic property calculations. Table 4 lists the calculated results of lead-free DJ OIHPs, with the imaginary part of phonon DOS, the Debye temperature, and the band gap data. For the [4-(azaniumylmethyl)-2-fluorophenyl]methanaminium PbI_4_ structure, Mg^2+^, Sr^2+^, Zn^2+^, and Yb^2+^ replacement of Pb^2+^ could decrease the imaginary part of phonon DOS from 0.71% to 0.33%, 0.25%, 0.09%, and 0.31%, respectively. Mg^2+^ and Yb^2+^ are the two cations that could obtain a direct band gap during the replacement. For the [4-(azaniumylmethyl)-2,6-difluorophenyl]methanaminium PbI_4_, the Mg^2+^, Zn^2+^, Eu^2+^, and Yb^2+^ replacements of Pb^2+^ could decrease the imaginary part of the phonon DOS from 0.69% to 0.35%, 0.04%, 0.21%, and 0.48%, respectively. Mg^2+^ and Eu^2+^ are the two cations that can provide direct band gaps during the replacement. The Mg^2+^ cation replacement of Pb^2+^ could change the band gap from the indirect band gap to direct in DJ OIHP. The Mg^2+^, Zn^2+^, and Yb^2+^ replacements of Pb^2^ could improve the dynamic stability in DJ OIHP. The [4-(azaniumylmethyl)-2-fluorophenyl]methanaminium ZnI_4_ and [4-(azaniumylmethyl)-2,6-difluorophenyl]methanaminium ZnI_4_ are the dynamic stable structures with the ultra-low imaginary part of the phonon DOS of 0.09% and 0.04%, and indirect band gaps as 4.00 eV and 3.81 eV, respectively. Even increasing the *k* point setting from 4 × 5 × 3 to 7 × 7 × 4, the imaginary part of the phonon DOS of [4-(azaniumylmethyl)-2-fluorophenyl]methanaminium ZnI_4_ and [4-(azaniumylmethyl)-2,6-difluorophenyl]methanaminium ZnI_4_ increased to 1.55% and 1.39%, respectively, remaining in lower amounts.

**Fig. 4:**
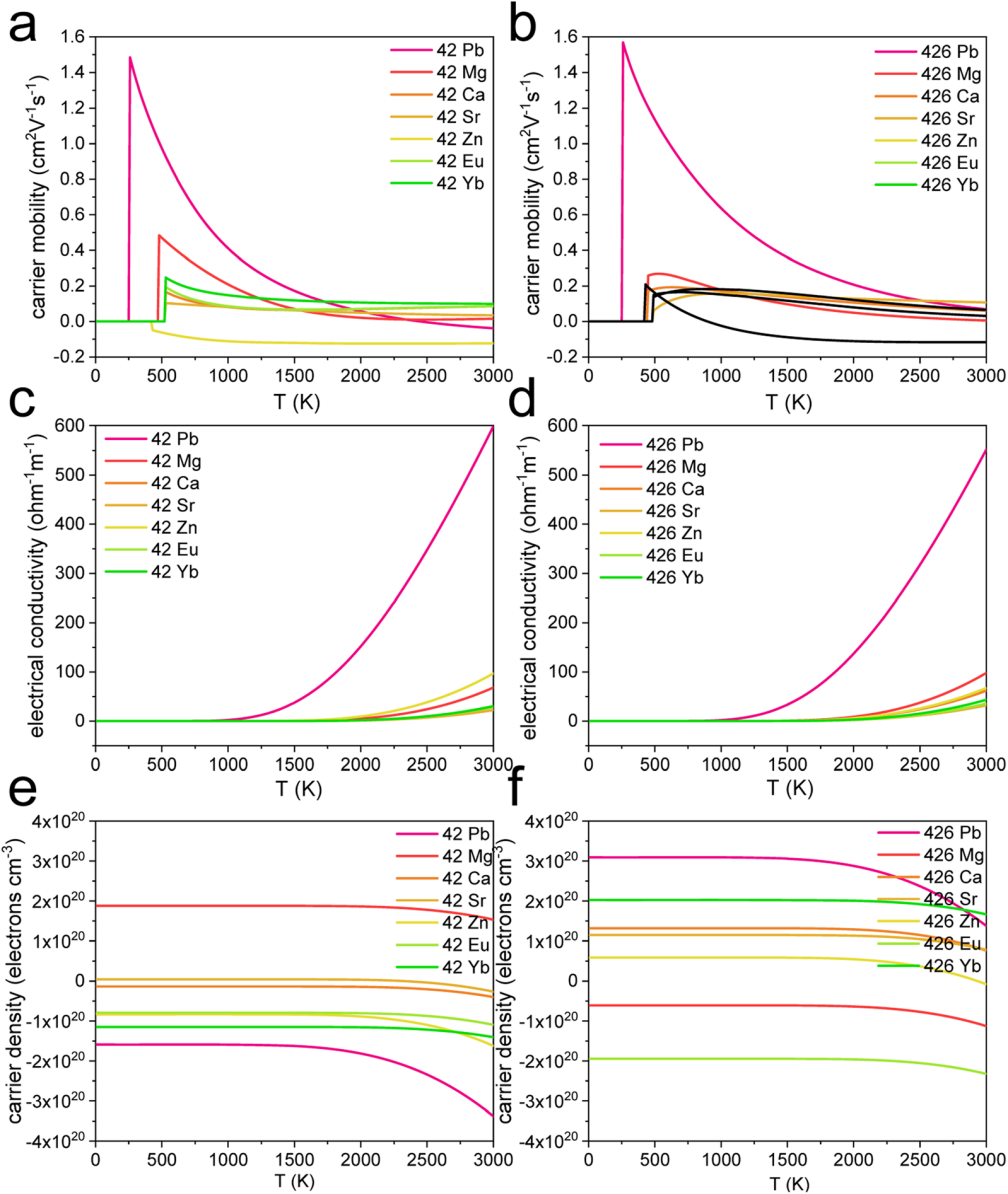
The carrier mobility, electrical conductivity, and carrier density of [4-(azaniumylmethyl)-2-fluorophenyl] methanaminium XI_4_ and [4-(azaniumylmethyl)-2,6-difluorophenyl] methanaminium XI_4_. (X = Pb, Mg, Ca, Sr, Zn, Eu, and Yb) (a and b) The carrier mobilities. (c and d) The electrical conductivities. (e and f) The carrier densities.

The properties of the lead-free DJ OIHPs should be thoroughly discussed for further application, such as carrier mobility, shown in Fig. 4a and b, electrical conductivity shown in Fig. 4c and d, and carrier density shown in Fig. 4e and f. Fig. 4a and b show that the Pb^2+^ cations inside the DJ OIHPs have the highest values for temperature-dependent carrier mobility. For the temperature-dependent electrical conductivity shown in Fig. 4c and d, the Pb^2+^ cations inside the DJ OIHPs have the highest values. In Fig. 4e, only the [4-(azaniumylmethyl)-2-fluorophenyl]methanaminium MgI_4_ is an n-type semiconductor with a positive value, and the others are p-type semiconductors. In Fig. 4f, only the [4-(azaniumylmethyl)-2,6-difluorophenyl]methanaminium MgI_4_ and [4-(azaniumylmethyl)-2,6-difluorophenyl]methanaminium EuI_4_ are p-type semiconductors, with the negative values, and the others are n-type semiconductors. The exact value of the carrier mobility and electrical conductivity for [4-(azaniumylmethyl)-2-fluorophenyl]methanaminium XI_4_ and [4-(azaniumylmethyl)-2,6-difluorophenyl]methanaminium XI_4_ are listed in Tables 3 and 4, respectively, and it was found that the lead-free DJ OIHPs became a semi-conductor at the higher temperature range. For the Zn-based lead-free DJ OIHPs, the working temperature should be higher than 450 K. As shown in Tables 3 and 4, the lead-containing DJ OIHPs have better optoelectronic properties than the lead-free ones and will be more suitable for device applications at room temperature. However, the lead-free DJ OIHPs have wider bands of 3.80 eV to 4.47 eV, as shown in Table 2, which is wider than the lead DJ OIHPs of 2.72 eV and are more stable. Therefore, the lead-free DJ OIHPs have the potential for wide-band gap device applications.

**Table 2:** The imaginary part of phonon DOS, Debye temperature, and band gap of [4-(azaniumylmethyl)-2-fluorophenyl] methanaminium XI_4_ and [4-(azaniumylmethyl)-2,6-difluorophenyl] methanaminium XI_4_. (X = Pb, Mg, Ca, Sr, Zn, Eu, and Yb)

[4-(Azaniumylmethyl)-2-fluorophenyl]methanaminium XI_4_	Imaginary part of phonon DOS (%)	Debye temperature (*θ*_D_) (K)	Band gap (eV)	[4-(Azaniumylmethyl)-2,6-difluorophenyl]methanaminium XI_4_	Imaginary part of phonon DOS (%)	Debye temperature (*θ*_D_) (K)	Band gap (eV)
X = Pb	0.71	101	2.72 (indirect)	X = Pb	0.69	94	2.72 (indirect)
X = Mg	0.33	116	4.03 (direct)	X = Mg	0.35	107	3.80 (direct)
X = Ca	0.90	103	4.36 (indirect)	X = Ca	1.17	103	4.02 (indirect)
X = Sr	0.25	97	4.47 (indirect)	X = Sr	3.67	114	4.08 (indirect)
X = Zn	0.09	108	4.00 (indirect)	X = Zn	0.04	111	3.81 (indirect)
X = Eu	0.90	102	4.44 (indirect)	X = Eu	0.21	105	4.09 (direct)
X = Yb	0.31	105	4.36 (direct)	X = Yb	0.48	104	4.03 (indirect)

**Table 3:** The electrical conductivity and carrier mobility of [4-(azaniumylmethyl)-2-fluorophenyl] methanaminium XI_4_. (X = Pb, Mg, Ca, Sr, Zn, Eu, and Yb) (*σ* (ohm^−1^ m^−1^) is electrical conductivity and *μ* (cm^2^ V^−1^ s^−1^) is the carrier mobility.)

[4-(Azaniumylmethyl)-2-fluorophenyl]methanaminium XI_4_	X = Pb		X = Mg		X = Ca		X = Sr		X = Zn		X = Eu		X = Yb	
	*σ*	*μ*	*σ*	*μ*	*σ*	*μ*	*σ*	*μ*	*σ*	*μ*	*σ*	*μ*	*σ*	*μ*
300 K	1.64 × 10^−8^	1.37	0	0	0	0	0	0	0	0	0	0	0	0
350 K	7.05 × 10^−7^	1.26	0	0	0	0	0	0	0	0	0	0	0	0
400 K	1.20 × 10^−5^	1.15	−2.56 × 10^−33^	0	0	0	0	0	0	0	0	0	0	0
450 K	1.10 × 10^−4^	1.06	−2.30 × 10^−31^	0	−4.78 × 10^−33^	0	−3.06 × 10^−33^	0	2.37 × 10^−10^	−0.05	−5.96 × 10^−33^	0	−5.46 × 10^−33^	0
500 K	6.53 × 10^−4^	0.97	3.44 × 10^−10^	0.47	−2.56 × 10^−31^	0	−1.71 × 10^−31^	0	5.44 × 10^−9^	−0.06	−3.01 × 10^−31^	0	−2.87 × 10^−31^	0
550 K	2.83 × 10^−3^	0.90	5.67 × 10^−9^	0.44	2.80 × 10^−10^	0.16	1.90 × 10^−10^	0.10	7.07 × 10^−8^	−0.07	3.23 × 10^−10^	0.18	3.11 × 10^−10^	0.24
600 K	9.63 × 10^−3^	0.82	5.89 × 10^−8^	0.41	3.72 × 10^−9^	0.14	2.55 × 10^−9^	0.10	6.02 × 10^−7^	−0.08	4.25 × 10^−9^	0.16	4.13 × 10^−9^	0.22

**Table 4:** The electrical conductivity and carrier mobility of [4-(azaniumylmethyl)-2,6-difluorophenyl]methanaminium XI_4_. (X = Pb, Mg, Ca, Sr, Zn, Eu, and Yb) (*σ* (ohm^−1^ m^−1^) is electrical conductivity and *μ* (cm^2^ V^−1^ s^−1^) is carrier mobility.)

[4-(Azaniumylmethyl)-2,6-difluorophenyl]methanaminium XI_4_	X = Pb		X = Mg		X = Ca		X = Sr		X = Zn		X = Eu		X = Yb	
	*σ*	*μ*	*σ*	*μ*	*σ*	*μ*	*σ*	*μ*	*σ*	*μ*	*σ*	*μ*	*σ*	*μ*
300 K	1.01 × 10^−8^	1.47	0	0	0	0	0	0	0	0	0	0	0	0
350 K	4.64 × 10^−7^	1.37	−2.77 × 10^−35^	0	−1.27 × 10^−35^	0	0	0	−1.58 × 10^−34^	0	0	0	0	0
400 K	8.33 × 10^−6^	1.28	−5.53 × 10^−32^	0	−4.77 × 10^−32^	0	−1.23 × 10^−33^	0	−6.53 × 10^−32^	0	−1.30 × 10^−33^	0	−1.27 × 10^−33^	0
450 K	7.96 × 10^−5^	1.21	1.41 × 10^−10^	0.26	1.21 × 10^−10^	0.17	−1.25 × 10^−31^	0	1.54 × 10^−10^	0.19	−1.28 × 10^−31^	0	−1.31 × 10^−31^	0
500 K	4.88 × 10^−4^	1.14	3.33 × 10^−9^	0.27	2.84 × 10^−9^	0.18	1.92 × 10^−10^	0.07	3.44 × 10^−9^	0.16	1.96 × 10^−10^	0.14	2.04 × 10^−10^	0.15
550 K	2.17 × 10^−3^	1.07	4.44 × 10^−8^	0.27	3.77 × 10^−8^	0.19	3.2 × 10^−9^	0.09	4.37 × 10^−8^	0.13	3.29 × 10^−9^	0.15	3.46 × 10^−9^	0.16
600 K	7.56 × 10^−3^	1.01	3.86 × 10^−7^	0.26	3.25 × 10^−7^	0.19	3.36 × 10^−8^	0.11	3.66 × 10^−7^	0.11	3.44 × 10^−8^	0.16	3.67 × 10^−8^	0.16

## Conclusions

By the phonon calculation, the fluoro-benzene-based divalent ammonium spacer cations could stabilize the DJ OIHP with increased dynamic stability. The Mg^2+^, Zn^2+^, and Yb^2+^ replacements of Pb^2+^ could improve the dynamic stability of the lead DJ OIHPs. Here, the [4-(azaniumylmethyl)-2-fluorophenyl]methanaminium ZnI_4_ (indirect, 4.00 eV) and [4-(azaniumylmethyl)-2,6-difluorophenyl]methanaminium ZnI_4_ (indirect, 3.81 eV) are the dynamic stable DJ OIHPs and might exist naturally with the imaginary part of phonon DOS as 0.09% and 0.04%. In this study, it was found that the two stable Zn-based lead-free DJ OIHPs could be used in future high-temperature ultra-wide band gap optoelectronic applications. Here, the [4-(azaniumylmethyl)-2-fluorophenyl]methanaminium ZnI_4_ (indirect, 4.00 eV) and [4-(azaniumylmethyl)-2,6-difluorophenyl]methanaminium ZnI_4_ (indirect, 3.81 eV) are the dynamic stable DJ OIHPs and might exist naturally with the imaginary part of phonon DOS as 0.09% and 0.04%, respectively. In this research, it was found that the two stable Zn-based lead-free DJ OIHPs could be used in future high-temperature ultra-wide band gap optoelectronic applications. Here, the [4-(azaniumylmethyl)-2-fluorophenyl]methanaminium ZnI_4_ (indirect, 4.00 eV) and [4-(azaniumylmethyl)-2,6-difluorophenyl]methanaminium ZnI_4_ (indirect, 3.81 eV) are the dynamic stable DJ OIHPs and might exist naturally with the imaginary part of phonon DOS as 0.09% and 0.04%, respectively. In this research, it was found that the two stable Zn-based lead-free DJ OIHPs could be used in future high-temperature ultra-wide band gap optoelectronic applications. Here, the [4-(azaniumylmethyl)-2-fluorophenyl]methanaminium ZnI_4_ (indirect, 4.00 eV) and [4-(azaniumylmethyl)-2,6-difluorophenyl]methanaminium ZnI_4_ (indirect, 3.81 eV) are the dynamic stable DJ OIHPs and might exist naturally with the imaginary part of phonon DOS as 0.09% and 0.04%, respectively. In this research, it was found that two stable Zn-based lead-free DJ OIHPs could be used in future high-temperature ultra-wide band gap optoelectronic applications. Here, the [4-(azaniumylmethyl)-2-fluorophenyl]methanaminium ZnI_4_ (indirect, 4.00 eV) and [4-(azaniumylmethyl)-2,6-difluorophenyl]methanaminium ZnI_4_ (indirect, 3.81 eV) are the dynamic stable DJ OIHPs and might exist naturally with the imaginary part of phonon DOS, as 0.09% and 0.04%, respectively. This research found that two stable Zn-based lead-free DJ OIHPs could be used in future high-temperature ultra-wide band gap optoelectronic applications. Here, the [4-(azaniumylmethyl)-2-fluorophenyl]methanaminium ZnI_4_ (indirect, 4.00 eV) and [4-(azaniumylmethyl)-2,6-difluorophenyl]methanaminium ZnI_4_ (indirect, 3.81 eV) are the dynamic stable DJ OIHPs and might exist naturally with the imaginary part of phonon DOS, as 0.09% and 0.04%, respectively. In this research, it was found that two stable Zn-based lead-free DJ OIHPs could be used in future high-temperature ultra-wide band gap optoelectronic applications. Here, the [4-(azaniumylmethyl)-2-fluorophenyl]methanaminium ZnI_4_ (indirect, 4.00 eV) and [4-(azaniumylmethyl)-2,6-difluorophenyl]methanaminium ZnI_4_ (indirect, 3.81 eV) are the dynamic stable DJ OIHPs and might exist naturally with the imaginary part of phonon DOS, as 0.09% and 0.04%, respectively. In this research, it was found that the two stable Zn-based lead-free DJ OIHPs could be used in future high-temperature ultra-wide band gap optoelectronic applications. Here, the [4-(azaniumylmethyl)-2-fluorophenyl]methanaminium ZnI_4_ (indirect, 4.00 eV) and [4-(azaniumylmethyl)-2,6-difluorophenyl]methanaminium ZnI_4_ (indirect, 3.81 eV) are the dynamic stable DJ OIHPs and might exist naturally with the imaginary part of phonon DOS, as 0.09% and 0.04%, respectively. In this research, it was found that two stable Zn-based lead-free DJ OIHPs could be used in future high-temperature ultra-wide band gap optoelectronic applications. Here, the [4-(azaniumylmethyl)-2-fluorophenyl]methanaminium ZnI_4_ (indirect, 4.00 eV) and [4-(azaniumylmethyl)-2,6-difluorophenyl]methanaminium ZnI_4_ (indirect, 3.81 eV) are the dynamic stable DJ OIHPs and might exist naturally with the imaginary part of phonon DOS, as 0.09% and 0.04%, respectively. In this research, it was found that the two stable Zn-based lead-free DJ OIHPs could be used in future high-temperature ultra-wide band gap optoelectronic applications.

## Method

The calculations were processed using VASP (Vienna *ab initio* Simulation Package)^21–23^ with different functionals for various applications. The new build-up structures were processed for structure optimization calculation using the GGA-PBEsol,^24^ with a default plane-wave cutoff energy of 500 eV. The requested *k*-spacing was 0.3 per Angstrom, which led to a 4 × 4 × 2 mesh, and using first-order Methfessel–Paxton smearing with a width of 0.2 eV. The phonon calculations were conducted using GGA-PBE,^25^ with a plane-wave cutoff of 500 eV, *k*-spacing of 4 × 5 × 3 and 7 × 7 × 4 mesh, and a Methfessel–Paxton smearing width of 0.2 eV. The 4 × 5 × 3 mesh was set for the first phonon state screen, and the 7 × 7 × 4 mesh was set for the detail phonon state discussion. The semiconductor properties (electrical conductivity, carrier mobility, and carrier density) were calculated within Boltzmann's transport theory (BoltzTraP), processed by GGA-PBEsol, with the chemical potential set as 18 mu within 42 functions. The chemical potential setup used MAPbCl_3_ as a reference structure, and 18 mu within 42 functions could obtain the calculation of electrical conductivity as 2.68 × 10^−6^ (ohm^−1^ m^−1^) at room temperature, which is close to the experimental result of 2.7 × 10^−6^ (ohm^−1^ m^−1^).^20^ The Meta GGA-MBJLDA^26,27^ was used for the band structure calculation with a plane-wave cutoff energy of 500 eV, *k*-spacing as 4 × 4 × 2 mesh, and linear-tetrahedron method. The XRD and electron diffraction pattern calculations were performed using Mercury and PTLab software.

## Author contributions

Chih Shan Tan initiated all the research ideas, performed calculations, and completed the paper writing independently.

## Conflicts of interest

The authors declare no competing financial interest.

## Supplementary Material

RA-013-D2RA07675F-s001
