# Supplementary material for: Stabile fluoro-benzene-based spacer for lead-free Dion–Jacobson perovskites
Source: RSC Adv. 2023 Jan 4;13(2):1185–93. doi: 10.1039/d2ra07675f (PMC9811935; doi:10.1039/d2ra07675f)
Supplement: RA-013-D2RA07675F-s001 [file RA-013-D2RA07675F-s001.pdf]

Electronic Supplementary Material (ESI) for RSC Advances.
This journal is © The Royal Society of Chemistry 2022

Supporting Information

Stabile Fluoro-benzene-based Lead-Free Dion-Jacobson Perovskite

Chih Shan Tan*

*Institute of Electronics, National Yang Ming Chiao Tung University, Hsinchu 30010, Taiwan

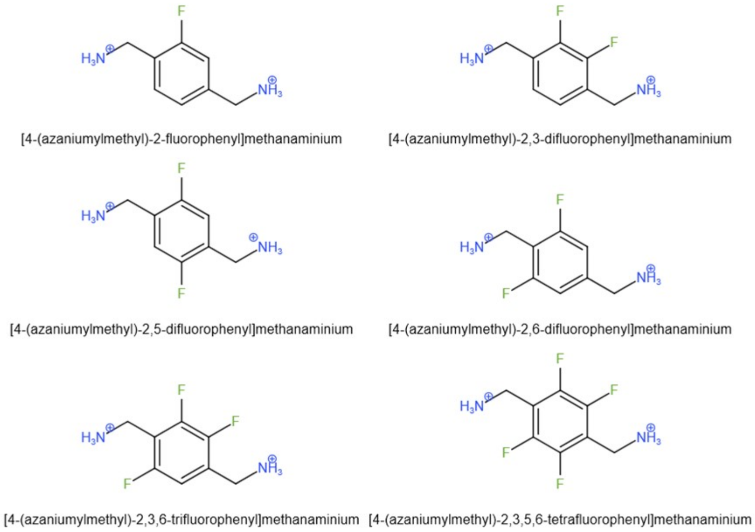

Figure S1. The different types of fluoro on [4-(azaniumylmethyl)phenyl]methanaminium spacer cations.

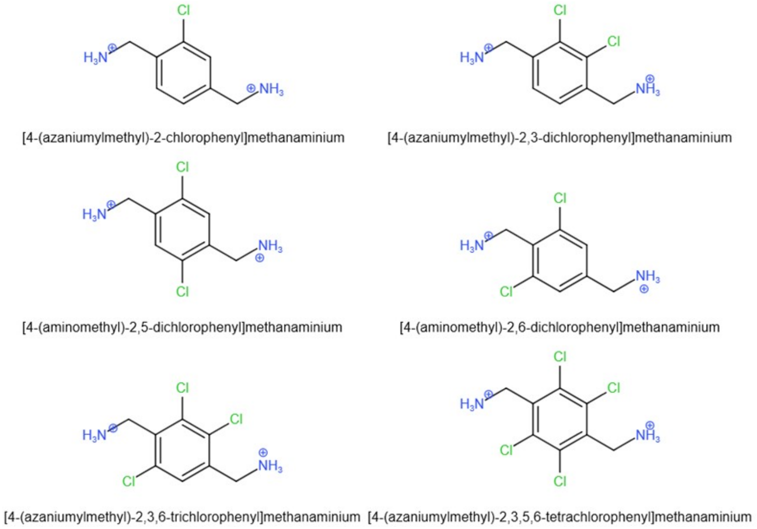

Figure S2. The different types of chloro on [4-(azaniumylmethyl)phenyl]methanaminium spacer cations.

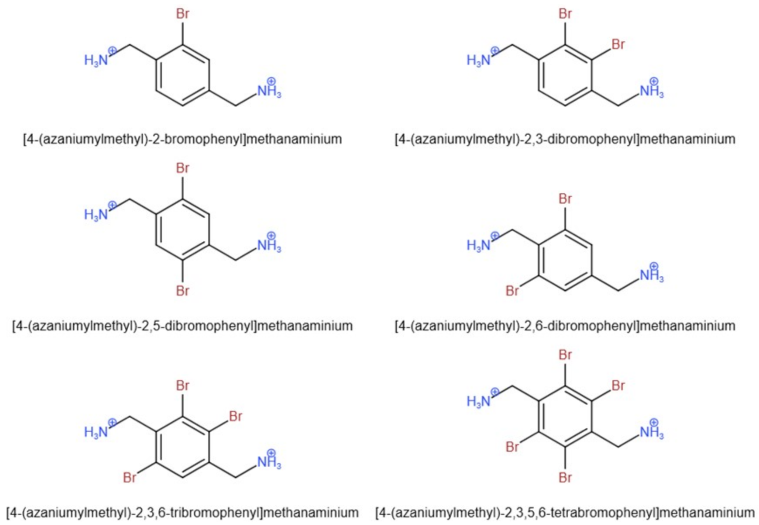

Figure S3. The different types of bromo on [4-(azaniumylmethyl)phenyl]methanaminium spacer cations.

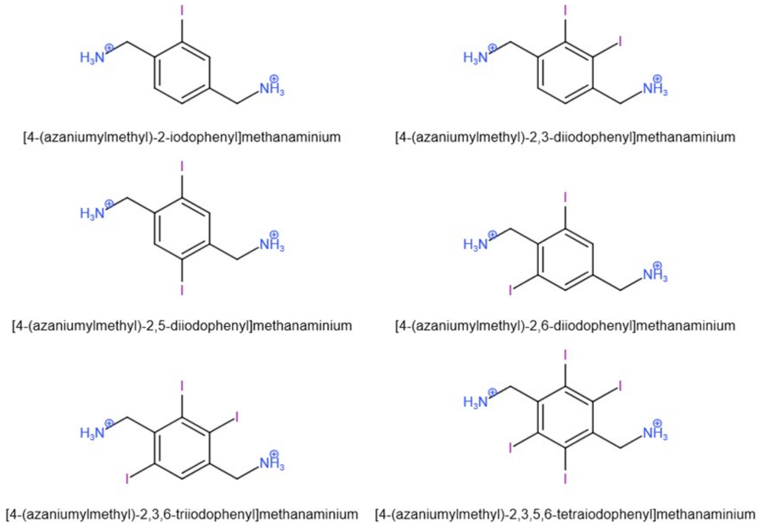

Figure S4. The different types of iodo on [4-(azaniumylmethyl)phenyl]methanaminium spacer cations.

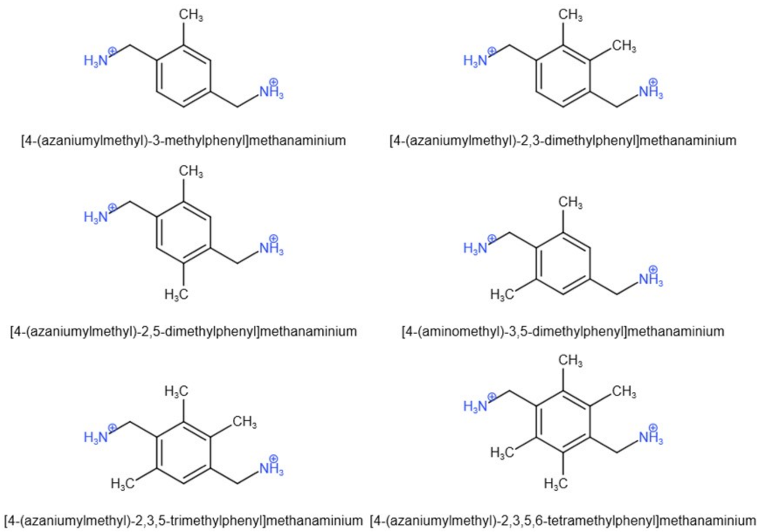

Figure S5. The different types of methyl on [4-(azaniumylmethyl)phenyl]methanaminium spacer cations.

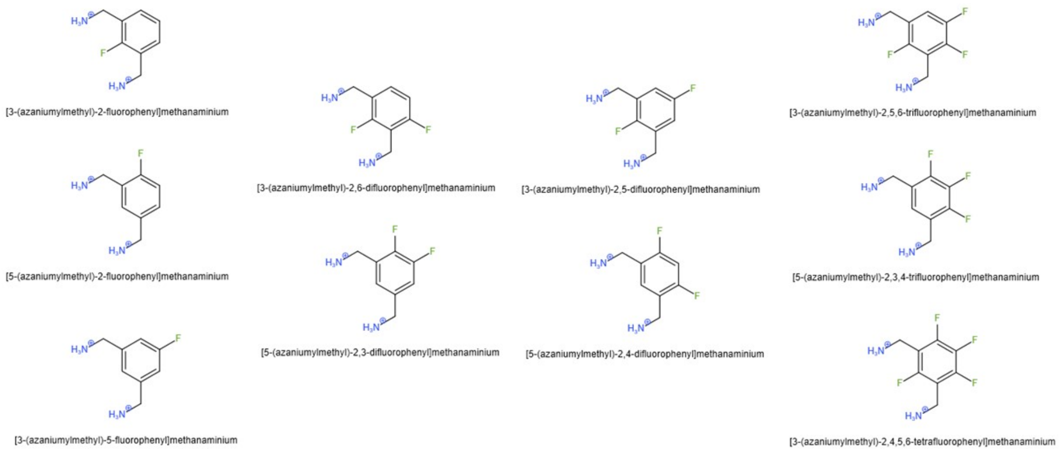

Figure S6. The different types of fluoro on [3-(azaniumylmethyl)phenyl]methanaminium spacer cations.

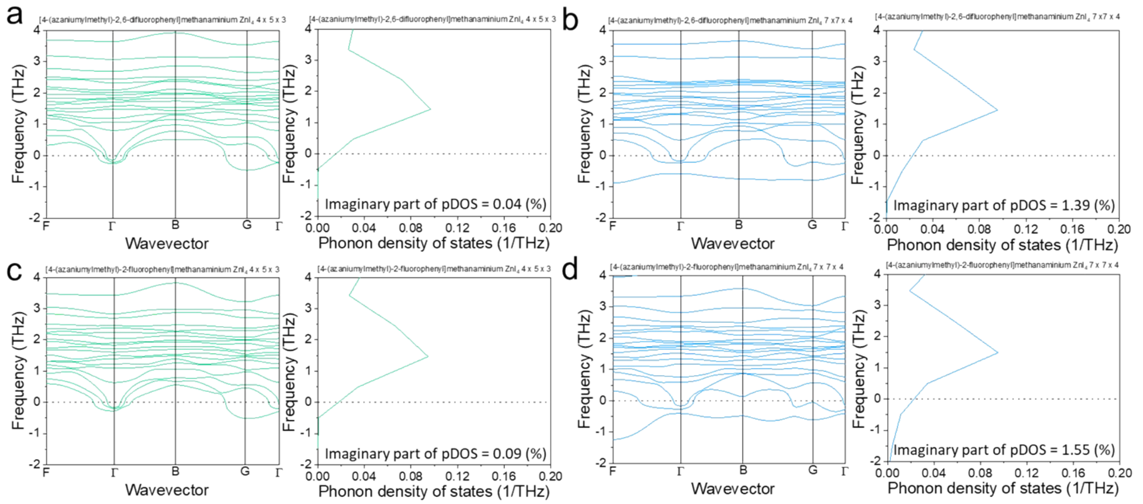

Figure S7. The different k-space settings of the [4-(azaniumylmethyl)-2,6-difluorophenyl]methanaminium ZnI_4 and [4-(azaniumylmethyl)-2-fluorophenyl]methanaminium ZnI_4. (a)-(b) The k-space settings as 4 x 5 x 3 and 7 x 7 x 4 for [4-(azaniumylmethyl)-2,6-difluorophenyl]methanaminium ZnI_4. (c)-(d) The k-space settings as 4 x 5 x 3 and 7 x 7 x 4 for [4-(azaniumylmethyl)-2-fluorophenyl]methanaminium ZnI_4.

Table S1. The imaginary part of phonon DOSs for different types of fluoro on [4-(azaniumylmethyl)phenyl]methanaminium PbI_4.

**Crystal Structure**	**Imaginary part of phonon DOS (%)**
[4-(azaniumylmethyl)-2-fluorophenyl]methanaminium PbI_4	0.71
[4-(azaniumylmethyl)-2,3-difluorophenyl]methanaminium PbI_4	1.82
[4-(azaniumylmethyl)-2,5-difluorophenyl]methanaminium PbI_4	1.92
[4-(azaniumylmethyl)-2,6-difluorophenyl]methanaminium PbI_4	0.69
[4-(azaniumylmethyl)-2,3,6-trifluorophenyl]methanaminium PbI_4	2.89
[4-(azaniumylmethyl)-2,3,5,6-tetrafluorophenyl]methanaminium PbI_4	1.98

Table S2. The imaginary part of phonon DOSs for different types of chloro on [4-(azaniumylmethyl)phenyl]methanaminium PbI_4.

Crystal Structure	Imaginary part of phonon DOS (%)
[4-(azaniumylmethyl)-2-chlorophenyl]methanaminium PbI_4	1.84
[4-(azaniumylmethyl)-2,3-dichlorophenyl]methanaminium PbI_4	1.31
[4-(azaniumylmethyl)-2,5-dichlorophenyl]methanaminium PbI_4	1.92
[4-(azaniumylmethyl)-2,6-dichlorophenyl]methanaminium PbI_4	1.56
[4-(azaniumylmethyl)-2,3,6-trichlorophenyl]methanaminium PbI_4	4.03
[4-(azaniumylmethyl)-2,3,5,6-tetrachlorophenyl]methanaminium PbI_4	1.55

Table S3. The imaginary part of phonon DOSs for different types of bromo on [4-(azaniumylmethyl)phenyl]methanaminium PbI_4.

Crystal Structure	Imaginary part of phonon DOS (%)
[4-(azaniumylmethyl)-2-bromophenyl]methanaminium PbI_4	1.54
[4-(azaniumylmethyl)-2,3-dibromophenyl]methanaminium PbI_4	2.72
[4-(azaniumylmethyl)-2,5-dibromophenyl]methanaminium PbI_4	2.40
[4-(azaniumylmethyl)-2,6-dibromophenyl]methanaminium PbI_4	1.44
[4-(azaniumylmethyl)-2,3,6-tribromophenyl]methanaminium PbI_4	1.22
[4-(azaniumylmethyl)-2,3,5,6-tetrabromophenyl]methanaminium PbI_4	3.18

Table S4. The imaginary part of phonon DOSs for different types of iodo on [4-(azaniumylmethyl)phenyl]methanaminium PbI_4.

Crystal Structure	Imaginary part of phonon DOS (%)
[4-(azaniumylmethyl)-2-iodophenyl]methanaminium PbI_4	1.30
[4-(azaniumylmethyl)-2,3-diiodophenyl]methanaminium PbI_4	2.55
[4-(azaniumylmethyl)-2,5-diiodophenyl]methanaminium PbI_4	1.74
[4-(azaniumylmethyl)-2,6-diiodophenyl]methanaminium PbI_4	2.88
[4-(azaniumylmethyl)-2,3,6-triiodophenyl]methanaminium PbI_4	2.68
[4-(azaniumylmethyl)-2,3,5,6-tetraiodophenyl]methanaminium PbI_4	1.40

Table S5. The imaginary part of phonon DOSs for different types of methyl on [4-(azaniumylmethyl)phenyl]methanaminium PbI_4.

Crystal Structure	Imaginary part of phonon DOS (%)
[4-(azaniumylmethyl)-3-methylphenyl]methanaminium PbI_4	2.19
[4-(azaniumylmethyl)-2,3-dimethylphenyl]methanaminium PbI_4	2.96
[4-(azaniumylmethyl)-2,5-dimethylphenyl]methanaminium PbI_4	2.04
[4-(azaniumylmethyl)-3,5-dimethylphenyl]methanaminium PbI_4	1.81
[4-(azaniumylmethyl)-2,3,5-trimethylphenyl]methanaminium PbI_4	2.82
[4-(azaniumylmethyl)-2,3,5,6-tetramethylphenyl]methanaminium PbI_4	3.64

Table S6. The imaginary part of phonon DOSs for different types of fluoro on [3-(azaniumylmethyl)phenyl]methanaminium PbI_4.

Crystal Structure	Imaginary part of phonon DOS (%)
[3-(azaniumylmethyl)phenyl]methanaminium PbI_4	1.44

[3-(azaniumylmethyl)-2-fluorophenyl]methanaminium PbI_4	2.76
[5-(azaniumylmethyl)-2-fluorophenyl]methanaminium PbI_4	3.35
[3-(azaniumylmethyl)-5-fluorophenyl]methanaminium PbI_4	2.44
[3-(azaniumylmethyl)-2,6-difluorophenyl]methanaminium PbI_4	1.56
[5-(azaniumylmethyl)-2,3-difluorophenyl]methanaminium PbI_4	2.24
[3-(azaniumylmethyl)-2,5-difluorophenyl]methanaminium PbI_4	2.76
[5-(azaniumylmethyl)-2,4-difluorophenyl]methanaminium PbI_4	1.36
[3-(azaniumylmethyl)-2,5,6-trifluorophenyl]methanaminium PbI_4	1.90
[5-(azaniumylmethyl)-2,3,4-trifluorophenyl]methanaminium PbI_4	1.74
[3-(azaniumylmethyl)-2,4,5,6-tetrafluorophenyl]methanaminium PbI_4	1.31

Crystal Information

==

Title [4-(azaniumylmethyl)phenyl]methanaminium PbI_4 ($C_8H_{14}I_4N_2Pb$)

Space group name *P1*

Lattice parameters

a	b	c	alpha	beta	gamma
6.43919	6.34323	12.67560	87.7300	89.9700	88.4800

Unit-cell volume = 517.149805 Å^3

Structure parameters

		x	y	z	Occ.	B	Site	Sym.
1 Pb	Pb_1	0.13507	0.93774	0.06847	1.000	1.000	1a	1
2 I	I_1	0.66479	0.06691	0.07497	1.000	1.000	1a	1
3 I	I_2	0.23846	0.90712	0.32306	1.000	1.000	1a	1
4 I	I_3	0.18379	0.44547	0.06863	1.000	1.000	1a	1
5 I	I_5	0.22057	0.94162	0.81743	1.000	1.000	1a	1
6 C	C_7	0.73422	0.43600	0.34147	1.000	1.000	1a	1

7 H	H_2	0.82366	0.29509	0.31877	1.000	1.000	1a	1
8 C	C_1	0.72163	0.45226	0.45920	1.000	1.000	1a	1
9 H	H_4	0.81957	0.79576	0.65275	1.000	1.000	1a	1
10 C	C_2	0.77549	0.63805	0.50686	1.000	1.000	1a	1
11 H	H_5	0.82246	0.77393	0.45775	1.000	1.000	1a	1
12 C	C_3	0.77425	0.65015	0.61669	1.000	1.000	1a	1
13 C	C_4	0.66544	0.27767	0.52359	1.000	1.000	1a	1
14 H	H_6	0.62297	0.13076	0.48853	1.000	1.000	1a	1
15 C	C_5	0.66449	0.28958	0.63322	1.000	1.000	1a	1
16 H	H_7	0.62208	0.15101	0.68145	1.000	1.000	1a	1
17 C	C_6	0.71910	0.47627	0.68106	1.000	1.000	1a	1
18 C	C_8	0.73388	0.48346	0.79935	1.000	1.000	1a	1
19 H	H_8	0.76987	0.64187	0.82396	1.000	1.000	1a	1
20 H	H_9	0.85693	0.37351	0.82993	1.000	1.000	1a	1
21 H	H_{11}	0.80565	0.57561	0.30477	1.000	1.000	1a	1
22 N	N_1	0.52224	0.41991	0.29237	1.000	1.000	1a	1
23 H	H_{12}	0.53170	0.40037	0.21154	1.000	1.000	1a	1
24 H	H_{13}	0.43104	0.55691	0.30407	1.000	1.000	1a	1
25 H	H_{14}	0.44216	0.28984	0.32232	1.000	1.000	1a	1
26 N	N_2	0.53605	0.42175	0.85427	1.000	1.000	1a	1
27 H	H_{15}	0.46176	0.29880	0.81866	1.000	1.000	1a	1
28 H	H_{16}	0.43063	0.54829	0.85660	1.000	1.000	1a	1
29 H	H_{17}	0.56576	0.36559	0.93153	1.000	1.000	1a	1

===

Title 2-[4-(2-azaniumylethyl)phenyl]ethan-1-aminium PbI_4 ($C_{10}H_{18}I_4N_2Pb$)

Space group name *P_1*

Lattice parameters

a	b	c	alpha	beta	gamma

6.50496 6.38672 13.44780 89.1100 90.4000 90.3600

Unit-cell volume = 558.602008 Å^3

Structure parameters

		x	y	z	Occ.	B	Site	Sym.
1 Pb	Pb_1	0.16571	0.89493	0.04960	1.000	1.000	1a	1
2 I	I_1	0.67619	0.87482	0.05462	1.000	1.000	1a	1
3 I	I_2	0.22785	0.88882	0.27663	1.000	1.000	1a	1
4 I	I_3	0.09948	0.39651	0.04334	1.000	1.000	1a	1
5 I	I_5	0.24768	0.91051	0.82199	1.000	1.000	1a	1
6 C	C_7	0.78456	0.38250	0.33110	1.000	1.000	1a	1
7 H	H_2	0.86510	0.23847	0.30844	1.000	1.000	1a	1
8 C	C_1	0.77960	0.39528	0.44274	1.000	1.000	1a	1
9 H	H_4	0.89343	0.73279	0.63023	1.000	1.000	1a	1
10 C	C_2	0.84089	0.57833	0.49048	1.000	1.000	1a	1
11 H	H_5	0.89379	0.71262	0.44548	1.000	1.000	1a	1
12 C	C_3	0.84080	0.58934	0.59391	1.000	1.000	1a	1
13 C	C_4	0.71287	0.22500	0.50118	1.000	1.000	1a	1
14 H	H_6	0.66215	0.08013	0.46541	1.000	1.000	1a	1
15 C	C_5	0.71287	0.23599	0.60437	1.000	1.000	1a	1
16 H	H_7	0.66233	0.09970	0.64870	1.000	1.000	1a	1
17 C	C_6	0.77957	0.41775	0.65232	1.000	1.000	1a	1
18 C	C_8	0.78935	0.42452	0.76412	1.000	1.000	1a	1
19 H	H_8	0.86091	0.57317	0.78811	1.000	1.000	1a	1
20 H	H_9	0.88937	0.29611	0.79231	1.000	1.000	1a	1
21 H	H_{11}	0.87290	0.51698	0.30013	1.000	1.000	1a	1
22 C	C_9	0.56875	0.38418	0.28806	1.000	1.000	1a	1
23 H	H_{13}	0.48789	0.53176	0.30308	1.000	1.000	1a	1
24 H	H_{14}	0.47402	0.25167	0.31544	1.000	1.000	1a	1

25 C	C_{10}	0.57794	0.39888	0.80959	1.000	1.000	1a	1
26 H	H_{15}	0.49750	0.25757	0.78304	1.000	1.000	1a	1
27 H	H_{16}	0.47888	0.53579	0.79506	1.000	1.000	1a	1
28 N	N_1	0.59383	0.37258	0.92073	1.000	1.000	1a	1
29 N	N_2	0.57486	0.36445	0.17764	1.000	1.000	1a	1
30 H	H_{18}	0.52183	0.23409	0.94379	1.000	1.000	1a	1
31 H	H_{19}	0.67899	0.47095	0.14664	1.000	1.000	1a	1
32 H	H_{20}	0.61642	0.21354	0.15633	1.000	1.000	1a	1
33 H	H_{21}	0.43029	0.39464	0.14601	1.000	1.000	1a	1
34 H	H_{22}	0.74680	0.36550	0.94486	1.000	1.000	1a	1
35 H	H_{23}	0.52619	0.49707	0.95666	1.000	1.000	1a	1

==

Title benzene-1,4-bis(aminium) PbI_4 ($C_6H_{10}I_4N_2Pb$)

Space group name *P_1*

Lattice parameters

a	b	c	alpha	beta	gamma
6.20957	6.35044	12.04020	87.5700	90.0700	89.8800

Unit-cell volume = 474.358860 Å^3

Structure parameters

		x	y	z	Occ.	B	Site	Sym.
1 Pb	Pb_1	0.19415	0.25002	0.12706	1.000	1.000	1a	1
2 I	I_1	0.69469	0.25850	0.11732	1.000	1.000	1a	1
3 I	I_2	0.18819	0.22079	0.39271	1.000	1.000	1a	1
4 I	I_3	0.20870	0.75057	0.13624	1.000	1.000	1a	1
5 I	I_5	0.17461	0.27084	0.86235	1.000	1.000	1a	1
6 N	N_3	0.69293	0.45860	0.38292	1.000	1.000	1a	1
7 H	H_2	0.82669	0.36331	0.36080	1.000	1.000	1a	1
8 H	H_3	0.54779	0.38811	0.35616	1.000	1.000	1a	1

9 C	C_1	0.68848	0.47802	0.50259	1.000	1.000	1a	1
10 H	H_4	0.72890	0.83752	0.70213	1.000	1.000	1a	1
11 C	C_2	0.71328	0.67270	0.54870	1.000	1.000	1a	1
12 H	H_5	0.73986	0.81361	0.49507	1.000	1.000	1a	1
13 C	C_3	0.70707	0.68580	0.66395	1.000	1.000	1a	1
14 C	C_4	0.65830	0.29500	0.56832	1.000	1.000	1a	1
15 H	H_6	0.63644	0.14387	0.52962	1.000	1.000	1a	1
16 C	C_5	0.65206	0.30817	0.68297	1.000	1.000	1a	1
17 H	H_7	0.62480	0.16797	0.73695	1.000	1.000	1a	1
18 C	C_6	0.67608	0.50367	0.72933	1.000	1.000	1a	1
19 N	N_2	0.67464	0.50932	0.84991	1.000	1.000	1a	1
20 H	H_8	0.64979	0.65817	0.87876	1.000	1.000	1a	1
21 H	H_{10}	0.54731	0.41418	0.88268	1.000	1.000	1a	1
22 H	H_9	0.82411	0.44969	0.88212	1.000	1.000	1a	1
23 H	H_{11}	0.71066	0.60082	0.33907	1.000	1.000	1a	1

==

Title [4-(azaniumylmethyl)-2,6-difluorophenyl]methanaminium CaI_4 ($C_8H_{12}CaF_2I_4N_2$)

Space group name *P_1*

Lattice parameters

a	b	c	alpha	beta	gamma
6.21259	6.05990	12.77985	88.6700	92.4900	83.6100

Unit-cell volume = 477.500455 Å^3

Structure parameters

		x	y	z	Occ.	B	Site	Sym.
1 Ca	Ca_1	0.13517	0.98937	0.05997	1.000	1.000	1a	1
2 I	I_1	0.62980	0.05956	0.06619	1.000	1.000	1a	1
3 I	I_2	0.18417	0.93821	0.29593	1.000	1.000	1a	1
4 I	I_3	0.08170	0.49620	0.05825	1.000	1.000	1a	1

5 I	I_5	0.16640	0.97711	0.81690	1.000	1.000	1a	1
6 C	C_7	0.69265	0.40791	0.35521	1.000	1.000	1a	1
7 H	H_2	0.79271	0.25024	0.33936	1.000	1.000	1a	1
8 C	C_1	0.67099	0.43361	0.47067	1.000	1.000	1a	1
9 H	H_4	0.83020	0.75491	0.67102	1.000	1.000	1a	1
10 C	C_2	0.75657	0.60321	0.52535	1.000	1.000	1a	1
11 C	C_3	0.75574	0.61963	0.63348	1.000	1.000	1a	1
12 C	C_4	0.58109	0.27937	0.53350	1.000	1.000	1a	1
13 C	C_5	0.57529	0.28690	0.64136	1.000	1.000	1a	1
14 H	H_7	0.50307	0.15589	0.68361	1.000	1.000	1a	1
15 C	C_6	0.66561	0.45915	0.69218	1.000	1.000	1a	1
16 C	C_8	0.67919	0.46373	0.80951	1.000	1.000	1a	1
17 H	H_8	0.74054	0.61689	0.83716	1.000	1.000	1a	1
18 H	H_9	0.79199	0.32149	0.84175	1.000	1.000	1a	1
19 H	H_{11}	0.77262	0.54561	0.32230	1.000	1.000	1a	1
20 N	N_1	0.48149	0.40465	0.29532	1.000	1.000	1a	1
21 H	H_{12}	0.50898	0.37229	0.21686	1.000	1.000	1a	1
22 H	H_{13}	0.38255	0.55790	0.29827	1.000	1.000	1a	1
23 H	H_{14}	0.39457	0.27462	0.31989	1.000	1.000	1a	1
24 N	N_2	0.46700	0.44815	0.85690	1.000	1.000	1a	1
25 H	H_{15}	0.38662	0.31652	0.82848	1.000	1.000	1a	1
26 H	H_{16}	0.36286	0.59700	0.84508	1.000	1.000	1a	1
27 H	H_{17}	0.48849	0.41297	0.93733	1.000	1.000	1a	1
28 F	F_1	0.84883	0.75710	0.47029	1.000	1.000	1a	1
29 F	F_2	0.49714	0.11055	0.48448	1.000	1.000	1a	1

==

Title [4-(azaniumylmethyl)-2,6-difluorophenyl]methanaminium EuI_4 ($C_8H_{12}EuF_2I_4N_2$)

Space group name *P_1*

Lattice parameters

a	b	c	alpha	beta	gamma
6.25596	6.14799	12.80899	88.4300	92.4100	82.4200

Unit-cell volume = 487.647028 Å^3

Structure parameters

		x	y	z	Occ.	B	Site	Sym.
1 Eu	Eu_1	0.13809	0.99897	0.06042	1.000	1.000	1a	1
2 I	I_1	0.62987	0.07986	0.06926	1.000	1.000	1a	1
3 I	I_2	0.19330	0.93646	0.30075	1.000	1.000	1a	1
4 I	I_3	0.07160	0.50846	0.05619	1.000	1.000	1a	1
5 I	I_5	0.17699	0.98162	0.81357	1.000	1.000	1a	1
6 C	C_7	0.69148	0.40578	0.35547	1.000	1.000	1a	1
7 H	H_2	0.79411	0.25039	0.33944	1.000	1.000	1a	1
8 C	C_1	0.67197	0.43108	0.47080	1.000	1.000	1a	1
9 H	H_4	0.82667	0.74724	0.66969	1.000	1.000	1a	1
10 C	C_2	0.75556	0.59768	0.52481	1.000	1.000	1a	1
11 C	C_3	0.75439	0.61398	0.63274	1.000	1.000	1a	1
12 C	C_4	0.58475	0.27944	0.53388	1.000	1.000	1a	1
13 C	C_5	0.57871	0.28717	0.64146	1.000	1.000	1a	1
14 H	H_7	0.50841	0.15836	0.68398	1.000	1.000	1a	1
15 C	C_6	0.66595	0.45686	0.69167	1.000	1.000	1a	1
16 C	C_8	0.67725	0.46147	0.80872	1.000	1.000	1a	1
17 H	H_8	0.73681	0.61168	0.83630	1.000	1.000	1a	1
18 H	H_9	0.78980	0.31966	0.84163	1.000	1.000	1a	1
19 H	H_{11}	0.76670	0.54221	0.32175	1.000	1.000	1a	1
20 N	N_1	0.48028	0.40189	0.29726	1.000	1.000	1a	1
21 H	H_{12}	0.50666	0.36756	0.21904	1.000	1.000	1a	1
22 H	H_{13}	0.38053	0.55444	0.29967	1.000	1.000	1a	1

23 H	H_{14}	0.39698	0.27459	0.32365	1.000	1.000	1a	1
24 N	N_2	0.46489	0.44932	0.85504	1.000	1.000	1a	1
25 H	H_{15}	0.38617	0.32106	0.82621	1.000	1.000	1a	1
26 H	H_{16}	0.36145	0.59867	0.84306	1.000	1.000	1a	1
27 H	H_{17}	0.48520	0.41350	0.93540	1.000	1.000	1a	1
28 F	F_1	0.84576	0.74826	0.46930	1.000	1.000	1a	1
29 F	F_2	0.50370	0.11306	0.48545	1.000	1.000	1a	1

==

Title [4-(azaniumylmethyl)-2,6-difluorophenyl]methanaminium MgI_4 ($C_8H_{12}F_2I_4MgN_2$)

Space group name P_1

Lattice parameters

a	b	c	alpha	beta	gamma
6.21416	5.83385	12.49943	89.0100	92.1900	85.4100

Unit-cell volume = 451.258031 Å^3

Structure parameters

		x	y	z	Occ.	B	Site	Sym.
1 Mg	Mg_1	0.09031	0.97227	0.05862	1.000	1.000	1a	1
2 I	I_1	0.63093	0.01530	0.06073	1.000	1.000	1a	1
3 I	I_2	0.17135	0.94038	0.28023	1.000	1.000	1a	1
4 I	I_3	0.08897	0.47121	0.06235	1.000	1.000	1a	1
5 I	I_5	0.14638	0.97069	0.83178	1.000	1.000	1a	1
6 C	C_7	0.69766	0.41116	0.35012	1.000	1.000	1a	1
7 H	H_2	0.79219	0.24698	0.33447	1.000	1.000	1a	1
8 C	C_1	0.67063	0.43838	0.46805	1.000	1.000	1a	1
9 H	H_4	0.83957	0.76776	0.67492	1.000	1.000	1a	1
10 C	C_2	0.76124	0.61284	0.52516	1.000	1.000	1a	1
11 C	C_3	0.75980	0.62891	0.63572	1.000	1.000	1a	1
12 C	C_4	0.57311	0.27977	0.53146	1.000	1.000	1a	1

13 C	C_5	0.56665	0.28654	0.64189	1.000	1.000	1a	1
14 H	H_7	0.48966	0.15072	0.68431	1.000	1.000	1a	1
15 C	C_6	0.66356	0.46309	0.69503	1.000	1.000	1a	1
16 C	C_8	0.68144	0.46726	0.81488	1.000	1.000	1a	1
17 H	H_8	0.74830	0.62608	0.84284	1.000	1.000	1a	1
18 H	H_9	0.79181	0.32059	0.84597	1.000	1.000	1a	1
19 H	H_{11}	0.78674	0.55153	0.31821	1.000	1.000	1a	1
20 N	N_1	0.49078	0.41000	0.28587	1.000	1.000	1a	1
21 H	H_{12}	0.52229	0.37651	0.20609	1.000	1.000	1a	1
22 H	H_{13}	0.39570	0.56819	0.28777	1.000	1.000	1a	1
23 H	H_{14}	0.39609	0.27653	0.30850	1.000	1.000	1a	1
24 N	N_2	0.47213	0.44983	0.86655	1.000	1.000	1a	1
25 H	H_{15}	0.38745	0.31172	0.83976	1.000	1.000	1a	1
26 H	H_{16}	0.36926	0.60207	0.85622	1.000	1.000	1a	1
27 H	H_{17}	0.49685	0.41632	0.94852	1.000	1.000	1a	1
28 F	F_1	0.86035	0.77220	0.47013	1.000	1.000	1a	1
29 F	F_2	0.48283	0.10588	0.48046	1.000	1.000	1a	1

==

Title [4-(azaniumylmethyl)-2,6-difluorophenyl]methanaminium PbI_4 ($C_8H_{12}F_2I_4N_2Pb$)

Space group name *P1*

Lattice parameters

a	b	c	alpha	beta	gamma
6.31684	6.17396	12.67527	88.1000	92.7200	83.8200

Unit-cell volume = 490.543502 Å^3

Structure parameters

		x	y	z	Occ.	B	Site	Sym.
1 Pb	Pb_1	0.11893	0.99342	0.06036	1.000	1.000	1a	1
2 I	I_1	0.61682	0.05486	0.06904	1.000	1.000	1a	1

3 I	I_2	0.16782	0.94617	0.30550	1.000	1.000	1a	1
4 I	I_3	0.08157	0.49876	0.06177	1.000	1.000	1a	1
5 I	I_5	0.16569	0.97060	0.80523	1.000	1.000	1a	1
6 C	C_7	0.66356	0.42193	0.35072	1.000	1.000	1a	1
7 H	H_2	0.76066	0.26827	0.33365	1.000	1.000	1a	1
8 C	C_1	0.64805	0.44524	0.46740	1.000	1.000	1a	1
9 H	H_4	0.78802	0.77328	0.66655	1.000	1.000	1a	1
10 C	C_2	0.72390	0.61751	0.52104	1.000	1.000	1a	1
11 C	C_3	0.72346	0.63431	0.63002	1.000	1.000	1a	1
12 C	C_4	0.57022	0.28765	0.53204	1.000	1.000	1a	1
13 C	C_5	0.56778	0.29400	0.64079	1.000	1.000	1a	1
14 H	H_7	0.50564	0.16014	0.68455	1.000	1.000	1a	1
15 C	C_6	0.64589	0.47014	0.69052	1.000	1.000	1a	1
16 C	C_8	0.65787	0.47551	0.80889	1.000	1.000	1a	1
17 H	H_8	0.70295	0.63281	0.83592	1.000	1.000	1a	1
18 H	H_9	0.77877	0.34550	0.84268	1.000	1.000	1a	1
19 H	H_{11}	0.73899	0.55862	0.31621	1.000	1.000	1a	1
20 N	N_1	0.45189	0.41842	0.29349	1.000	1.000	1a	1
21 H	H_{12}	0.47048	0.39483	0.21330	1.000	1.000	1a	1
22 H	H_{13}	0.35403	0.56678	0.30000	1.000	1.000	1a	1
23 H	H_{14}	0.37105	0.28712	0.31875	1.000	1.000	1a	1
24 N	N_2	0.45186	0.44060	0.85563	1.000	1.000	1a	1
25 H	H_{15}	0.37974	0.30973	0.82388	1.000	1.000	1a	1
26 H	H_{16}	0.34344	0.58270	0.84584	1.000	1.000	1a	1
27 H	H_{17}	0.47467	0.39871	0.93627	1.000	1.000	1a	1
28 F	F_1	0.80509	0.77369	0.46377	1.000	1.000	1a	1
29 F	F_2	0.49442	0.11750	0.48383	1.000	1.000	1a	1

===

Title [4-(azaniumylmethyl)-2,6-difluorophenyl]methanaminium SrI_4 ($C_8H_{12}F_2I_4N_2Sr$)

Space group name *P1*

Lattice parameters

a	b	c	alpha	beta	gamma
6.38675	6.23673	12.92211	88.0000	91.9800	80.3400

Unit-cell volume = 506.685054 Å^3

Structure parameters

		x	y	z	Occ.	B	Site	Sym.
1 Sr	Sr_1	0.14642	0.01077	0.06322	1.000	1.000	1a	1
2 I	I_1	0.63065	0.11484	0.07235	1.000	1.000	1a	1
3 I	I_2	0.20594	0.93844	0.30733	1.000	1.000	1a	1
4 I	I_3	0.05880	0.52777	0.05058	1.000	1.000	1a	1
5 I	I_5	0.19262	0.99592	0.81262	1.000	1.000	1a	1
6 C	C_7	0.68969	0.40095	0.35700	1.000	1.000	1a	1
7 H	H_2	0.79578	0.24794	0.33999	1.000	1.000	1a	1
8 C	C_1	0.67381	0.42434	0.47144	1.000	1.000	1a	1
9 H	H_4	0.82021	0.73400	0.66677	1.000	1.000	1a	1
10 C	C_2	0.75459	0.58653	0.52399	1.000	1.000	1a	1
11 C	C_3	0.75216	0.60337	0.63084	1.000	1.000	1a	1
12 C	C_4	0.58907	0.27721	0.53464	1.000	1.000	1a	1
13 C	C_5	0.58192	0.28571	0.64109	1.000	1.000	1a	1
14 H	H_7	0.51266	0.16173	0.68409	1.000	1.000	1a	1
15 C	C_6	0.66522	0.45129	0.68991	1.000	1.000	1a	1
16 C	C_8	0.67261	0.45734	0.80584	1.000	1.000	1a	1
17 H	H_8	0.73043	0.60357	0.83257	1.000	1.000	1a	1
18 H	H_9	0.78234	0.31367	0.83963	1.000	1.000	1a	1
19 H	H_{11}	0.75620	0.53744	0.32255	1.000	1.000	1a	1
20 N	N_1	0.48089	0.39755	0.30227	1.000	1.000	1a	1

21 H	H_{12}	0.50557	0.36201	0.22446	1.000	1.000	1a	1
22 H	H_{13}	0.37987	0.54986	0.30496	1.000	1.000	1a	1
23 H	H_{14}	0.40435	0.27244	0.33075	1.000	1.000	1a	1
24 N	N_2	0.46195	0.45428	0.85080	1.000	1.000	1a	1
25 H	H_{15}	0.38490	0.33072	0.82247	1.000	1.000	1a	1
26 H	H_{16}	0.36133	0.60594	0.83837	1.000	1.000	1a	1
27 H	H_{17}	0.48069	0.41792	0.93066	1.000	1.000	1a	1
28 F	F_1	0.84243	0.73213	0.46793	1.000	1.000	1a	1
29 F	F_2	0.51093	0.11505	0.48747	1.000	1.000	1a	1

==

Title [4-(azaniumylmethyl)-2,6-difluorophenyl]methanaminium YbI_4 ($C_8H_{12}F_2I_4N_2Yb$)

Space group name P_1

Lattice parameters

a	b	c	alpha	beta	gamma
6.14921	6.01844	12.80526	88.7200	92.6300	84.4100

Unit-cell volume = 470.983564 Å^3

Structure parameters

		x	y	z	Occ.	B	Site	Sym.
1 Yb	Yb_1	0.13160	0.98631	0.05857	1.000	1.000	1a	1
2 I	I_1	0.62815	0.04492	0.06373	1.000	1.000	1a	1
3 I	I_2	0.17928	0.93695	0.29298	1.000	1.000	1a	1
4 I	I_3	0.08475	0.49101	0.05818	1.000	1.000	1a	1
5 I	I_5	0.15974	0.97479	0.81780	1.000	1.000	1a	1
6 C	C_7	0.69451	0.40890	0.35601	1.000	1.000	1a	1
7 H	H_2	0.79378	0.25029	0.34067	1.000	1.000	1a	1
8 C	C_1	0.67084	0.43501	0.47117	1.000	1.000	1a	1
9 H	H_4	0.83346	0.75918	0.67169	1.000	1.000	1a	1
10 C	C_2	0.75771	0.60643	0.52610	1.000	1.000	1a	1

11 C	C_3	0.75704	0.62253	0.63405	1.000	1.000	1a	1
12 C	C_4	0.57867	0.27923	0.53356	1.000	1.000	1a	1
13 C	C_5	0.57290	0.28644	0.64123	1.000	1.000	1a	1
14 H	H_7	0.49912	0.15396	0.68313	1.000	1.000	1a	1
15 C	C_6	0.66530	0.46013	0.69235	1.000	1.000	1a	1
16 C	C_8	0.68098	0.46447	0.80950	1.000	1.000	1a	1
17 H	H_8	0.74573	0.61845	0.83699	1.000	1.000	1a	1
18 H	H_9	0.79354	0.32145	0.84117	1.000	1.000	1a	1
19 H	H_{11}	0.77863	0.54695	0.32390	1.000	1.000	1a	1
20 N	N_1	0.48237	0.40591	0.29487	1.000	1.000	1a	1
21 H	H_{12}	0.51054	0.37597	0.21662	1.000	1.000	1a	1
22 H	H_{13}	0.38372	0.55947	0.29796	1.000	1.000	1a	1
23 H	H_{14}	0.39289	0.27403	0.31801	1.000	1.000	1a	1
24 N	N_2	0.46734	0.44852	0.85736	1.000	1.000	1a	1
25 H	H_{15}	0.38479	0.31485	0.82981	1.000	1.000	1a	1
26 H	H_{16}	0.36302	0.59753	0.84530	1.000	1.000	1a	1
27 H	H_{17}	0.48896	0.41584	0.93772	1.000	1.000	1a	1
28 F	F_1	0.85180	0.76248	0.47165	1.000	1.000	1a	1
29 F	F_2	0.49285	0.10872	0.48453	1.000	1.000	1a	1

==

Title [4-(azaniumylmethyl)-2,6-difluorophenyl]methanaminium ZnI_4 ($C_8H_{12}F_2I_4N_2Zn$)

Space group name *P1*

Lattice parameters

a	b	c	alpha	beta	gamma
6.57698	6.23080	11.73803	89.3900	91.0000	81.7400

Unit-cell volume = 475.918177 Å^3

Structure parameters

	x	y	z	Occ.	B	Site	Sym.

1	Zn	Zn_1	0.05474	0.07422	0.06436	1.000	1.000	1a	1
2	I	I_1	0.66781	0.02818	0.06245	1.000	1.000	1a	1
3	I	I_2	0.20485	0.94295	0.26590	1.000	1.000	1a	1
4	I	I_3	0.07402	0.48471	0.07287	1.000	1.000	1a	1
5	I	I_5	0.18013	0.97459	0.85398	1.000	1.000	1a	1
6	C	C_7	0.69058	0.40503	0.33330	1.000	1.000	1a	1
7	H	H_2	0.78627	0.24931	0.31363	1.000	1.000	1a	1
8	C	C_1	0.67131	0.43168	0.45933	1.000	1.000	1a	1
9	H	H_4	0.82304	0.73754	0.67781	1.000	1.000	1a	1
10	C	C_2	0.75493	0.59212	0.51908	1.000	1.000	1a	1
11	C	C_3	0.75178	0.60881	0.63700	1.000	1.000	1a	1
12	C	C_4	0.58364	0.28587	0.52761	1.000	1.000	1a	1
13	C	C_5	0.57711	0.29331	0.64506	1.000	1.000	1a	1
14	H	H_7	0.50947	0.16753	0.69118	1.000	1.000	1a	1
15	C	C_6	0.66252	0.45766	0.70068	1.000	1.000	1a	1
16	C	C_8	0.67150	0.46273	0.82825	1.000	1.000	1a	1
17	H	H_8	0.72675	0.61060	0.85979	1.000	1.000	1a	1
18	H	H_9	0.77854	0.32205	0.86223	1.000	1.000	1a	1
19	H	H_{11}	0.76517	0.53547	0.29541	1.000	1.000	1a	1
20	N	N_1	0.49058	0.40399	0.27173	1.000	1.000	1a	1
21	H	H_{12}	0.51958	0.35071	0.18813	1.000	1.000	1a	1
22	H	H_{13}	0.39941	0.55757	0.26858	1.000	1.000	1a	1
23	H	H_{14}	0.40459	0.28937	0.30445	1.000	1.000	1a	1
24	N	N_2	0.46980	0.45071	0.88103	1.000	1.000	1a	1
25	H	H_{15}	0.39274	0.33040	0.84668	1.000	1.000	1a	1
26	H	H_{16}	0.37150	0.59997	0.87605	1.000	1.000	1a	1
27	H	H_{17}	0.49089	0.40354	0.96662	1.000	1.000	1a	1
28	F	F_1	0.84733	0.73581	0.45930	1.000	1.000	1a	1

29 F	F_2	0.50345	0.12430	0.47412	1.000	1.000	1a	1

==

Title [4-(azaniumylmethyl)-2-fluorophenyl]methanaminium CaI_4 ($C_8H_{13}CaFI_4N_2$)

Space group name P_1

Lattice parameters

a	b	c	alpha	beta	gamma
6.20919	6.08132	12.52340	90.4300	92.8600	85.0200

Unit-cell volume = 470.510174 Å^3

Structure parameters

		x	y	z	Occ.	B	Site	Sym.
1 Ca	Ca_1	0.17306	0.00681	0.06151	1.000	1.000	1a	1
2 I	I_1	0.66972	0.06538	0.06171	1.000	1.000	1a	1
3 I	I_2	0.23814	0.97378	0.30540	1.000	1.000	1a	1
4 I	I_3	0.12326	0.51220	0.06162	1.000	1.000	1a	1
5 I	I_5	0.19898	0.98523	0.81486	1.000	1.000	1a	1
6 C	C_7	0.73420	0.43074	0.34371	1.000	1.000	1a	1
7 H	H_2	0.82282	0.27136	0.32318	1.000	1.000	1a	1
8 C	C_1	0.70890	0.44742	0.46196	1.000	1.000	1a	1
9 H	H_4	0.84786	0.77709	0.67279	1.000	1.000	1a	1
10 C	C_2	0.78215	0.62409	0.52074	1.000	1.000	1a	1
11 C	C_3	0.78055	0.63700	0.63135	1.000	1.000	1a	1
12 C	C_4	0.62448	0.28094	0.51970	1.000	1.000	1a	1
13 H	H_6	0.56490	0.13897	0.47647	1.000	1.000	1a	1
14 C	C_5	0.61893	0.29039	0.63036	1.000	1.000	1a	1
15 H	H_7	0.55424	0.15539	0.67299	1.000	1.000	1a	1
16 C	C_6	0.69980	0.46731	0.68722	1.000	1.000	1a	1
17 C	C_8	0.71332	0.46985	0.80719	1.000	1.000	1a	1
18 H	H_8	0.78432	0.61887	0.83864	1.000	1.000	1a	1

19 H	H_9	0.81599	0.32447	0.83850	1.000	1.000	1a	1
20 H	H_{11}	0.82534	0.56420	0.31499	1.000	1.000	1a	1
21 N	N_1	0.52267	0.44233	0.28158	1.000	1.000	1a	1
22 H	H_{12}	0.54610	0.41169	0.20083	1.000	1.000	1a	1
23 H	H_{13}	0.43423	0.59708	0.28891	1.000	1.000	1a	1
24 H	H_{14}	0.42655	0.31732	0.30402	1.000	1.000	1a	1
25 N	N_2	0.49794	0.46344	0.85353	1.000	1.000	1a	1
26 H	H_{15}	0.41452	0.32993	0.82500	1.000	1.000	1a	1
27 H	H_{16}	0.39925	0.61125	0.83907	1.000	1.000	1a	1
28 H	H_{17}	0.51420	0.43942	0.93590	1.000	1.000	1a	1
29 F	F_1	0.86170	0.79044	0.46811	1.000	1.000	1a	1

==

Title [4-(azaniumylmethyl)-2-fluorophenyl]methanaminium EuI_4 ($C_8H_{13}EuFI_4N_2$)

Space group name *P_1*

Lattice parameters

a	b	c	alpha	beta	gamma
6.25229	6.17172	12.51366	90.2300	92.5200	84.3100

Unit-cell volume = 480.025549 Å^3

Structure parameters

		x	y	z	Occ.	B	Site	Sym.
1 Eu	Eu_1	0.17449	0.01240	0.06299	1.000	1.000	1a	1
2 I	I_1	0.66943	0.07870	0.06321	1.000	1.000	1a	1
3 I	I_2	0.24937	0.97488	0.31214	1.000	1.000	1a	1
4 I	I_3	0.12041	0.51909	0.06319	1.000	1.000	1a	1
5 I	I_5	0.20857	0.98542	0.81150	1.000	1.000	1a	1
6 C	C_7	0.73173	0.43134	0.34271	1.000	1.000	1a	1
7 H	H_2	0.82052	0.27480	0.32064	1.000	1.000	1a	1
8 C	C_1	0.71161	0.44560	0.46148	1.000	1.000	1a	1

9 H	H_4	0.84284	0.77196	0.67128	1.000	1.000	1a	1
10 C	C_2	0.78068	0.62053	0.51973	1.000	1.000	1a	1
11 C	C_3	0.77904	0.63313	0.63040	1.000	1.000	1a	1
12 C	C_4	0.63258	0.27979	0.51973	1.000	1.000	1a	1
13 H	H_6	0.57758	0.13851	0.47707	1.000	1.000	1a	1
14 C	C_5	0.62693	0.28885	0.63047	1.000	1.000	1a	1
15 H	H_7	0.56665	0.15458	0.67364	1.000	1.000	1a	1
16 C	C_6	0.70243	0.46456	0.68678	1.000	1.000	1a	1
17 C	C_8	0.71153	0.46766	0.80673	1.000	1.000	1a	1
18 H	H_8	0.77921	0.61439	0.83848	1.000	1.000	1a	1
19 H	H_9	0.81343	0.32372	0.83888	1.000	1.000	1a	1
20 H	H_{11}	0.81801	0.56417	0.31281	1.000	1.000	1a	1
21 N	N_1	0.51818	0.44368	0.28408	1.000	1.000	1a	1
22 H	H_{12}	0.53641	0.41455	0.20274	1.000	1.000	1a	1
23 H	H_{13}	0.42965	0.59676	0.29307	1.000	1.000	1a	1
24 H	H_{14}	0.42579	0.32018	0.30849	1.000	1.000	1a	1
25 N	N_2	0.49516	0.46222	0.85139	1.000	1.000	1a	1
26 H	H_{15}	0.41409	0.33219	0.82133	1.000	1.000	1a	1
27 H	H_{16}	0.39759	0.60942	0.83684	1.000	1.000	1a	1
28 H	H_{17}	0.50786	0.43599	0.93372	1.000	1.000	1a	1
29 F	F_1	0.85634	0.78528	0.46634	1.000	1.000	1a	1

==

Title [4-(azaniumylmethyl)-2-fluorophenyl]methanaminium MgI_4 ($C_8H_{13}FI_4MgN_2$)

Space group name P_1

Lattice parameters

a	b	c	alpha	beta	gamma
6.12628	5.85715	12.38213	90.3000	92.8900	86.6100

Unit-cell volume = 442.959592 Å^3

Structure parameters

		x	y	z	Occ.	B	Site	Sym.
1 Mg	Mg_1	0.14792	0.98585	0.06085	1.000	1.000	1a	1
2 I	I_1	0.67092	0.02102	0.06019	1.000	1.000	1a	1
3 I	I_2	0.21937	0.96757	0.28833	1.000	1.000	1a	1
4 I	I_3	0.12486	0.48697	0.06295	1.000	1.000	1a	1
5 I	I_5	0.17957	0.97768	0.83106	1.000	1.000	1a	1
6 C	C_7	0.73993	0.43415	0.34164	1.000	1.000	1a	1
7 H	H_2	0.82955	0.26974	0.32410	1.000	1.000	1a	1
8 C	C_1	0.70421	0.45459	0.46023	1.000	1.000	1a	1
9 H	H_4	0.85309	0.79616	0.67346	1.000	1.000	1a	1
10 C	C_2	0.78214	0.63794	0.51990	1.000	1.000	1a	1
11 C	C_3	0.77993	0.65180	0.63153	1.000	1.000	1a	1
12 C	C_4	0.61046	0.28450	0.51879	1.000	1.000	1a	1
13 H	H_6	0.54589	0.13732	0.47523	1.000	1.000	1a	1
14 C	C_5	0.60445	0.29444	0.63084	1.000	1.000	1a	1
15 H	H_7	0.53466	0.15444	0.67375	1.000	1.000	1a	1
16 C	C_6	0.69390	0.47621	0.68849	1.000	1.000	1a	1
17 C	C_8	0.71736	0.47805	0.80953	1.000	1.000	1a	1
18 H	H_8	0.79023	0.63538	0.83975	1.000	1.000	1a	1
19 H	H_9	0.82629	0.33137	0.83879	1.000	1.000	1a	1
20 H	H_{11}	0.83763	0.57272	0.31413	1.000	1.000	1a	1
21 N	N_1	0.53309	0.44106	0.27311	1.000	1.000	1a	1
22 H	H_{12}	0.56581	0.40765	0.19272	1.000	1.000	1a	1
23 H	H_{13}	0.44344	0.59973	0.27715	1.000	1.000	1a	1
24 H	H_{14}	0.43017	0.31097	0.29323	1.000	1.000	1a	1
25 N	N_2	0.50665	0.46121	0.86250	1.000	1.000	1a	1
26 H	H_{15}	0.41856	0.32153	0.83545	1.000	1.000	1a	1

27 H	H_{16}	0.40431	0.61127	0.85192	1.000	1.000	1a	1
28 H	H_{17}	0.53357	0.43337	0.94518	1.000	1.000	1a	1
29 F	F_1	0.87016	0.80967	0.46703	1.000	1.000	1a	1

===

Title [4-(azaniumylmethyl)-2-fluorophenyl]methanaminium PbI_4 ($C_8H_{13}FI_4N_2Pb$)

Space group name P_1

Lattice parameters

a	b	c	alpha	beta	gamma
6.28778	6.17496	12.53183	88.5300	92.0200	86.4300

Unit-cell volume = 485.135543 Å^3

Structure parameters

		x	y	z	Occ.	B	Site	Sym.
1 Pb	Pb_1	0.18505	0.97325	0.06745	1.000	1.000	1a	1
2 I	I_1	0.68193	0.03383	0.06926	1.000	1.000	1a	1
3 I	I_2	0.25618	0.93655	0.32007	1.000	1.000	1a	1
4 I	I_3	0.15034	0.47774	0.06580	1.000	1.000	1a	1
5 I	I_5	0.23417	0.94850	0.81245	1.000	1.000	1a	1
6 C	C_7	0.73425	0.43037	0.34076	1.000	1.000	1a	1
7 H	H_2	0.83016	0.28242	0.32004	1.000	1.000	1a	1
8 C	C_1	0.71940	0.44578	0.45940	1.000	1.000	1a	1
9 H	H_4	0.82550	0.79725	0.65842	1.000	1.000	1a	1
10 C	C_2	0.77205	0.63231	0.51188	1.000	1.000	1a	1
11 C	C_3	0.77463	0.64846	0.62213	1.000	1.000	1a	1
12 C	C_4	0.65917	0.27191	0.52302	1.000	1.000	1a	1
13 H	H_6	0.61660	0.12227	0.48471	1.000	1.000	1a	1
14 C	C_5	0.65776	0.28407	0.63340	1.000	1.000	1a	1
15 H	H_7	0.61157	0.14386	0.68081	1.000	1.000	1a	1
16 C	C_6	0.71830	0.47154	0.68396	1.000	1.000	1a	1

17 C	C_8	0.73455	0.47752	0.80354	1.000	1.000	1a	1
18 H	H_8	0.77822	0.63772	0.83066	1.000	1.000	1a	1
19 H	H_9	0.85775	0.35501	0.83554	1.000	1.000	1a	1
20 H	H_{11}	0.80779	0.57221	0.30626	1.000	1.000	1a	1
21 N	N_1	0.51994	0.41945	0.28730	1.000	1.000	1a	1
22 H	H_{12}	0.53083	0.39911	0.20550	1.000	1.000	1a	1
23 H	H_{13}	0.42462	0.56325	0.29838	1.000	1.000	1a	1
24 H	H_{14}	0.43979	0.28445	0.31434	1.000	1.000	1a	1
25 N	N_2	0.53068	0.42925	0.85402	1.000	1.000	1a	1
26 H	H_{15}	0.45728	0.29703	0.82066	1.000	1.000	1a	1
27 H	H_{16}	0.42176	0.56579	0.84827	1.000	1.000	1a	1
28 H	H_{17}	0.55687	0.38396	0.93444	1.000	1.000	1a	1
29 F	F_1	0.82566	0.80591	0.45288	1.000	1.000	1a	1

==

Title [4-(azaniumylmethyl)-2-fluorophenyl]methanaminium SrI_4 ($C_8H_{13}FI_4N_2Sr$)

Space group name *P_1*

Lattice parameters

a	b	c	alpha	beta	gamma
6.38512	6.26574	12.52770	90.2900	92.1700	82.1600

Unit-cell volume = 496.161114 Å^3

Structure parameters

		x	y	z	Occ.	B	Site	Sym.
1 Sr	Sr_1	0.18717	0.02186	0.06344	1.000	1.000	1a	1
2 I	I_1	0.67807	0.10443	0.06497	1.000	1.000	1a	1
3 I	I_2	0.25746	0.98188	0.31867	1.000	1.000	1a	1
4 I	I_3	0.12042	0.53187	0.06343	1.000	1.000	1a	1
5 I	I_5	0.22309	0.99244	0.80637	1.000	1.000	1a	1
6 C	C_7	0.72503	0.43037	0.34232	1.000	1.000	1a	1

7 H	H_2	0.81618	0.27625	0.31786	1.000	1.000	1a	1
8 C	C_1	0.71238	0.44164	0.46131	1.000	1.000	1a	1
9 H	H_4	0.83417	0.76223	0.67041	1.000	1.000	1a	1
10 C	C_2	0.77736	0.61306	0.51913	1.000	1.000	1a	1
11 C	C_3	0.77634	0.62506	0.62976	1.000	1.000	1a	1
12 C	C_4	0.64090	0.27786	0.51963	1.000	1.000	1a	1
13 H	H_6	0.58979	0.13947	0.47697	1.000	1.000	1a	1
14 C	C_5	0.63574	0.28647	0.63027	1.000	1.000	1a	1
15 H	H_7	0.57863	0.15500	0.67341	1.000	1.000	1a	1
16 C	C_6	0.70471	0.45959	0.68616	1.000	1.000	1a	1
17 C	C_8	0.70848	0.46424	0.80590	1.000	1.000	1a	1
18 H	H_8	0.76803	0.60975	0.83767	1.000	1.000	1a	1
19 H	H_9	0.81096	0.32219	0.83959	1.000	1.000	1a	1
20 H	H_{11}	0.80193	0.56281	0.31118	1.000	1.000	1a	1
21 N	N_1	0.51211	0.44296	0.28799	1.000	1.000	1a	1
22 H	H_{12}	0.52766	0.41188	0.20653	1.000	1.000	1a	1
23 H	H_{13}	0.42314	0.59592	0.29740	1.000	1.000	1a	1
24 H	H_{14}	0.42631	0.32246	0.31479	1.000	1.000	1a	1
25 N	N_2	0.49424	0.46099	0.84862	1.000	1.000	1a	1
26 H	H_{15}	0.41936	0.33272	0.81835	1.000	1.000	1a	1
27 H	H_{16}	0.39650	0.60810	0.83321	1.000	1.000	1a	1
28 H	H_{17}	0.50516	0.43541	0.93098	1.000	1.000	1a	1
29 F	F_1	0.84680	0.77544	0.46547	1.000	1.000	1a	1

==

Title [4-(azaniumylmethyl)-2-fluorophenyl]methanaminium YbI_4 ($C_8H_{13}FI_4N_2Yb$)

Space group name *P_1*

Lattice parameters

a b c alpha beta gamma

6.14698 6.03799 12.54643 90.2800 92.9200 85.8200

Unit-cell volume = 463.823798 Å^3

Structure parameters

		x	y	z	Occ.	B	Site	Sym.
1 Yb	Yb_1	0.16986	0.00211	0.06122	1.000	1.000	1a	1
2 I	I_1	0.66762	0.05082	0.06152	1.000	1.000	1a	1
3 I	I_2	0.23409	0.97007	0.30295	1.000	1.000	1a	1
4 I	I_3	0.12446	0.50597	0.06180	1.000	1.000	1a	1
5 I	I_5	0.19348	0.98200	0.81676	1.000	1.000	1a	1
6 C	C_7	0.73655	0.43183	0.34429	1.000	1.000	1a	1
7 H	H_2	0.82486	0.27192	0.32484	1.000	1.000	1a	1
8 C	C_1	0.70802	0.44926	0.46213	1.000	1.000	1a	1
9 H	H_4	0.84866	0.78336	0.67197	1.000	1.000	1a	1
10 C	C_2	0.78131	0.62850	0.52058	1.000	1.000	1a	1
11 C	C_3	0.77983	0.64169	0.63096	1.000	1.000	1a	1
12 C	C_4	0.62136	0.28134	0.51994	1.000	1.000	1a	1
13 H	H_6	0.56099	0.13740	0.47703	1.000	1.000	1a	1
14 C	C_5	0.61563	0.29096	0.63051	1.000	1.000	1a	1
15 H	H_7	0.54982	0.15422	0.67301	1.000	1.000	1a	1
16 C	C_6	0.69864	0.46950	0.68703	1.000	1.000	1a	1
17 C	C_8	0.71504	0.47199	0.80673	1.000	1.000	1a	1
18 H	H_8	0.78835	0.62248	0.83776	1.000	1.000	1a	1
19 H	H_9	0.81894	0.32649	0.83762	1.000	1.000	1a	1
20 H	H_{11}	0.83087	0.56639	0.31610	1.000	1.000	1a	1
21 N	N_1	0.52485	0.44236	0.28067	1.000	1.000	1a	1
22 H	H_{12}	0.55049	0.41400	0.20030	1.000	1.000	1a	1
23 H	H_{13}	0.43552	0.59705	0.28793	1.000	1.000	1a	1
24 H	H_{14}	0.42631	0.31469	0.30186	1.000	1.000	1a	1

25 N	N_2	0.49911	0.46301	0.85450	1.000	1.000	1a	1
26 H	H_{15}	0.41370	0.32760	0.82685	1.000	1.000	1a	1
27 H	H_{16}	0.39957	0.61058	0.84036	1.000	1.000	1a	1
28 H	H_{17}	0.51772	0.43981	0.93669	1.000	1.000	1a	1
29 F	F_1	0.86245	0.79695	0.46792	1.000	1.000	1a	1

==

Title [4-(azaniumylmethyl)-2-fluorophenyl]methanaminium ZnI_4 ($C_8H_{13}FI_4N_2Zn$)

Space group name *P1*

Lattice parameters

a	b	c	alpha	beta	gamma
6.56797	6.10324	11.65676	90.2800	92.3600	84.3800

Unit-cell volume = 464.631034 Å^3

Structure parameters

		x	y	z	Occ.	B	Site	Sym.
1 Zn	Zn_1	0.08674	0.91278	0.06337	1.000	1.000	1a	1
2 I	I_1	0.69633	0.02838	0.06034	1.000	1.000	1a	1
3 I	I_2	0.23861	0.97627	0.27472	1.000	1.000	1a	1
4 I	I_3	0.12566	0.48294	0.06543	1.000	1.000	1a	1
5 I	I_5	0.20574	0.98389	0.85116	1.000	1.000	1a	1
6 C	C_7	0.72785	0.43509	0.32536	1.000	1.000	1a	1
7 H	H_2	0.81367	0.27735	0.30197	1.000	1.000	1a	1
8 C	C_1	0.70788	0.45282	0.45265	1.000	1.000	1a	1
9 H	H_4	0.84790	0.77942	0.67628	1.000	1.000	1a	1
10 C	C_2	0.78217	0.62668	0.51432	1.000	1.000	1a	1
11 C	C_3	0.78117	0.64186	0.63304	1.000	1.000	1a	1
12 C	C_4	0.62509	0.29135	0.51633	1.000	1.000	1a	1
13 H	H_6	0.56670	0.15024	0.47152	1.000	1.000	1a	1
14 C	C_5	0.62053	0.30307	0.63533	1.000	1.000	1a	1

15 H	H_7	0.55843	0.17077	0.68222	1.000	1.000	1a	1
16 C	C_6	0.70058	0.47758	0.69473	1.000	1.000	1a	1
17 C	C_8	0.71328	0.48347	0.82326	1.000	1.000	1a	1
18 H	H_8	0.77294	0.63572	0.85578	1.000	1.000	1a	1
19 H	H_9	0.81686	0.34333	0.85811	1.000	1.000	1a	1
20 H	H_{11}	0.81002	0.56847	0.29203	1.000	1.000	1a	1
21 N	N_1	0.52624	0.44395	0.26195	1.000	1.000	1a	1
22 H	H_{12}	0.54628	0.40465	0.17582	1.000	1.000	1a	1
23 H	H_{13}	0.44174	0.59916	0.26645	1.000	1.000	1a	1
24 H	H_{14}	0.43388	0.32443	0.28884	1.000	1.000	1a	1
25 N	N_2	0.51169	0.46989	0.87486	1.000	1.000	1a	1
26 H	H_{15}	0.43099	0.34365	0.84090	1.000	1.000	1a	1
27 H	H_{16}	0.41641	0.61885	0.86707	1.000	1.000	1a	1
28 H	H_{17}	0.53050	0.43075	0.96168	1.000	1.000	1a	1
29 F	F_1	0.86224	0.78760	0.45634	1.000	1.000	1a	1

===
